# Biochemical Investigations of Five Recombinantly Expressed Tyrosinases Reveal Two Novel Mechanisms Impacting Carbon Storage in Wetland Ecosystems

**DOI:** 10.1021/acs.est.3c02910

**Published:** 2023-09-01

**Authors:** Felix Panis, Annette Rompel

**Affiliations:** † Universität Wien, Fakultät für Chemie, Institut für Biophysikalische Chemie, Josef-Holaubek-Platz 2, 1090 Wien, Austria, https://www.bpc.univie.ac.at/en/

**Keywords:** global warming, climate change, carbon cycling, peatlands, enzymes, oxidoreductases, phenols

## Abstract

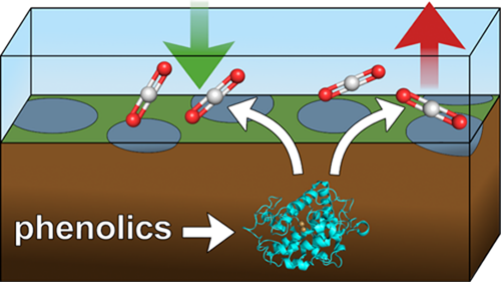

Wetlands are globally distributed ecosystems characterized by predominantly anoxic soils, resulting from water-logging. Over the past millennia, low decomposition rates of organic matter led to the accumulation of 20–30% of the world’s soil carbon pool in wetlands. Phenolic compounds are critically involved in stabilizing wetland carbon stores as they act as broad-scale inhibitors of hydrolytic enzymes. Tyrosinases are oxidoreductases capable of removing phenolic compounds in the presence of O_2_ by oxidizing them to the corresponding *o*-quinones. Herein, kinetic investigations (*k*_cat_ and *K*_m_ values) reveal that low-molecular-weight phenolic compounds naturally present within wetland ecosystems (including monophenols, diphenols, triphenols, and flavonoids) are accepted by five recombinantly expressed wetland tyrosinases (TYRs) as substrates. Investigations of the interactions between TYRs and wetland phenolics reveal two novel mechanisms that describe the global impact of TYRs on the wetland carbon cycle. First, it is shown that *o*-quinones (produced by TYRs from low-molecular-weight phenolic substrates) are capable of directly inactivating hydrolytic enzymes. Second, it is reported that *o*-quinones can interact with high-molecular-weight phenolic polymers (which inhibit hydrolytic enzymes) and remove them through precipitation. The balance between these two mechanisms will profoundly affect the fate of wetland carbon stocks, particularly in the wake of climate change.

## Abstract 2

Two novel mechanisms describing the impact of tyrosinases on carbon storage in wetland ecosystems have been identified using five recombinantly expressed tyrosinases produced by bacteria indigenous to globally distributed wetland ecosystems.

## Introduction

**Figure 1:**
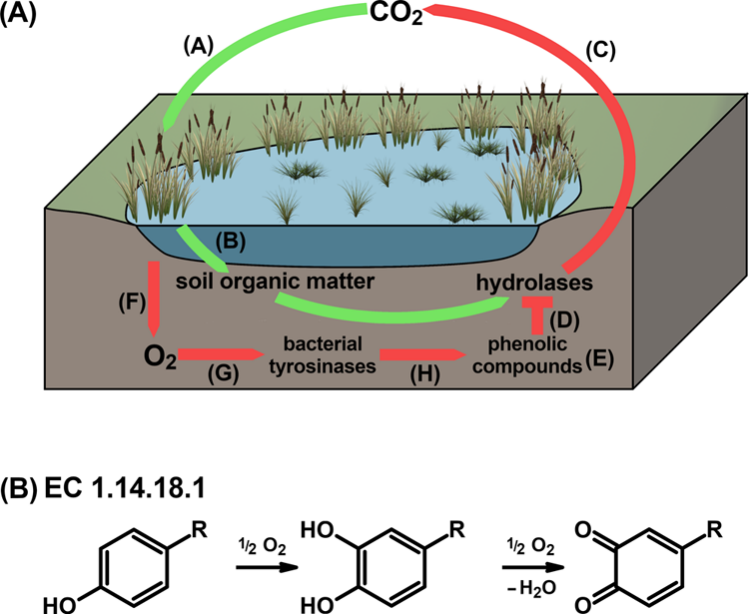
Schematic representation of the latch mechanism (A) and TYR activity (B). A: CO_2_ is converted into complex organic molecules via photosynthesis (A) and stored as soil organic matter (B), which originates predominantly from plant necromasses and plant litter. The degradation of soil organic carbon via hydrolases (C) is blocked (D) by a high concentration of phenolic compounds (E). Anoxic wetland soils (F) prevent the oxidative removal (G) of phenolic compounds by bacterial TYRs (H), thus leading to the accumulation of wetland carbon stores. B: TYRs catalyze the *o*-hydroxylation of monophenols to the corresponding *o*-diphenols and the subsequent two-electron oxidation of *o*-diphenols to the corresponding *o*-quinones. *o*-quinones are highly reactive, unstable molecules that spontaneously polymerize. When monophenols are converted into *o*-quinones, a characteristic lag period is required for TYRs to reach the maximum enzymatic activity.^26,27^ The figure has been created using GIMP 2.10.18 (https://www.gimp.org).

Wetlands are ecosystems characterized by permanently or seasonally water-logged soils in combination with plant growth.^1,2^ For most types of wetlands (peatlands, mangrove forests, bogs, and marshes), high levels of phenolic compounds have been reported.^3,4^ Wetlands represent unbalanced ecosystems in which the rate of carbon sequestration from the atmosphere (*via* the Calvin cycle of plant photosynthesis) exceeds the rate of carbon release, predominantly as CO_2_ and CH_4_.^5,6^ Thus, they have accumulated vast amounts of carbon over the last millennia. While wetlands cover only 5–8% of the terrestrial land surface, they are estimated to store 20–30% (500–550 × 10^15^ g) of the global soil carbon pool, which is equivalent to 66–72% of the entire atmospheric carbon pool of 760 × 10^15^ g.^2,7−9^ Within recent years, the so-called “latch mechanism” has been established to explain how wetlands act as long-term carbon sinks (Figure 1A). According to the “latch mechanism,” the imbalance between carbon storage and release results from the inhibition of organic matter degrading enzymes (*e.g.*, β-glucosidases, peroxidases, xylosidases, and chitinases)^10−12^ by phenolic compounds. Phenolic compounds act as unspecific enzyme inhibitors and are naturally abundant within wetland ecosystems^4,13^ due to plant secondary metabolism. Enzymes capable of oxidatively removing phenolic compounds in the presence of molecular oxygen (or H_2_O_2_ for peroxidases) are often grouped under the umbrella term “phenol oxidases”, and include, among others, tyrosinases, laccases, and peroxidases.^4^ In wetlands, the activity of phenol oxidases (and thus the removal of phenolic compounds) is restricted by oxygen scarcity, resulting from water-logging.^13^ Climate change, which will lead to increased temperatures and reduced rainfall, threatens water tables in wetlands and will, therefore, promote the aeration of previously anoxic wetland soils. This, in turn, will lead to increased levels of phenol oxidase activity, a consecutively reduced concentration of phenolic compounds, and an increased activity of organic matter degrading hydrolases.^13,14^ As a consequence, the stability of wetland carbon stores is at risk, and vast amounts of carbon will potentially be emitted back into the atmosphere, which itself will further promote climate change.

Ample experimental evidence supporting the central hypothesis of the “latch mechanism” (Figure 1A) has been published over the last years,^10,13−18^ however, contradictory observations led to the development of complementary mechanisms describing the processes controlling carbon cycling within wetland ecosystems.^19−23^ In a recent study, several enzyme groups (1,4-benzoquinone reductase, chalcone isomerase, flavanonol-cleaving reductase, phloretin hydrolase, phloroglucinol reductase, caffeoyl-CoA reductase, indole-pyruvate oxidoreductase, phenylacetate-CoA ligase, aromatic amino acid aminotransferase) involved in anoxic phenol metabolism in wetland soils have been identified, which are not affected by the “latch mechanism”.^19^ Furthermore, Wang et al. demonstrated in an alpine wetland that the enzymatic breakdown of soil organic matter and phenolic compounds is dependent on the relative concentrations of Fe(II) and Fe(III) ions, which potentially counteract the “latch mechanism”.^20^ Consequently, it was concluded that several mechanisms (“latch mechanism”, anoxic phenol metabolism, and Fe-dependent phenol metabolism) in concert control carbon cycling in wetland ecosystems.^24,25^

While numerous studies simulated the effects of climate change on carbon cycling within wetland ecosystems on a macroscopic scale,^10,13−18,20−23^ investigations focused on the precise enzymes controlling these processes are scarce. A recent review showed that a variety of bacteria (including Acidobacteria, Actinobacteria, Bacteroidetes, Firmicutes, Nitrospirae, Planctomycetes, and Proteobacteria) capable of producing tyrosinase enzymes (TYRs) are present in globally distributed wetland ecosystems (including peatlands, marshes, mangrove forests, bogs, and alkaline soda lakes).^4^ Thus, it has been concluded that TYRs are among the key enzymes controlling carbon cycling within wetland ecosystems (besides laccases, peroxidases, and enzymes involved in anoxic phenol metabolism).^4^ The relative impacts of TYRs, laccases, peroxidases, and enzymes involved in anoxic phenol metabolism on carbon cycling in wetland ecosystems require further investigation.

TYRs are type III copper proteins featuring a dicopper center and are ubiquitously distributed in nature among archaea,^28^ bacteria,^29^ fungi,^30^ plants,^31^ and animals,^32^ including humans.^33^ They catalyze the *o*-hydroxylation of monophenols to *o*-diphenols (EC 1.14.18.1), as well as the subsequent oxidation of *o*-diphenols to *o*-quinones (EC 1.10.3.1), which is coupled to the reduction of molecular oxygen to water (Figure 1B).^27^ Kinetic investigations of bacterial TYRs originating from non-wetland sources revealed that TYRs, in general, accept monophenols, diphenols, triphenols, and flavonoids as substrates with tyramine (monophenol), l-tyrosine (monophenol), dopamine (diphenol), and l-DOPA (diphenol) often used as standard substrates for testing mono- and diphenolase activities.^4^ Several phenolic compounds naturally abundant within wetland ecosystems (caffeic acid,^34,35^ catechin,^36^ epicatechin,^36^ gallic acid,^34^*p*-coumaric acid,^34,37^*p*-hydroxybenzoic acid,^34^ and protocatechuic acid,^34^Figure S1) have been reported to be accepted by bacterial TYRs as substrates.^4^ Structural investigations of TYRs revealed a conserved architecture of the active center while the overall folds of TYRs show little conservation.^4,38^ In the active center, two copper ions are coordinated by three histidine residues each (Figure S2).^39−41^ Second shell amino acids located in a distance of ∼5–15 Å around the active center are responsible for substrate selection and orientation and, therefore, govern the substrate scope and substrate preferences.^42−44^ Bacterial TYRs (except TYRs from *Streptomyces* and *Bacillus* species) are expressed in their latent states and require an activation step to develop catalytic activity. *In vivo*, this activation is proposedly achieved by an autolytic mechanism,^45^ which can be mimicked *in vitro* by the addition of suitable molarities of SDS.^46−48^

This study has been performed to straddle the previously disparate research areas of macroscopic investigations of carbon fluxes within wetland ecosystems and biochemical investigations of TYR enzymes. Biochemical investigations of five recombinantly expressed TYR enzymes (*CanS*TYR, *CabS*TYR, *SinA*TYR, *PseS*TYR, and *ChrS*TYR) identified within the genomes of bacteria indigenous to wetland ecosystems and originating from a phylogenetically diverse set of host organisms (including Acidobacteria, Planctomycetes, and Proteobacteria) showed that phenolic compounds naturally present within wetland ecosystems are commonly accepted as substrates. Furthermore, in-depth investigations of the interactions between *SinA*TYR and wetland phenolics (low-molecular-weight phenolics, humic acids, and lignin degradation products) have been performed. *SinA*TYR represents a planctomycetal TYR from *Singulisphaera acidiphila*, identified within a peatland in Russia. Importantly, these investigations revealed two novel and competing mechanisms that describe how TYRs impact the stability of organic carbon stored in wetland ecosystems. First, wetland carbon stores will be stabilized by increased TYR activity due to the inactivation of hydrolytic enzymes by quinones (produced from low-molecular-weight phenolic precursors, such as *p*-coumaric acid, caffeic acid, and catechin), which are naturally present within wetland ecosystems. Second, results presented herein demonstrate that high-molecular-weight phenolic polymers, responsible for the inhibition of hydrolytic enzymes, can be partially removed via precipitation as a result of increased TYR activity. Increased TYR activity in wetland soils has become a likely scenario in recent years due to climate change.^13,49,50^ The balance between the two novel mechanisms presented herein will impact the fate of wetland carbon stores and will, consequently, affect the future development of the global climate itself.

## Results and Discussion

### Sequence Selection, Expression, and Purification

TYRs have been selected from a phylogenetically diverse set of host organisms (Table 1) native to wetlands located in different climatic zones (Table 1) to verify that TYR enzymes affect the wetland carbon cycle on a global scale. This study constitutes the first investigation of proteobacterial, acidobacterial, and planctomycetal TYRs from host organisms indigenous to wetland ecosystems and the first investigation of acidobacterial and planctomycetal TYRs from any source.

**Table 1:** Recombinantly Expressed TYRs^a^

enzyme	host	phylum	UniProt IDs	location
*CanS*TYR	*Candidatus sulfopaludibacter* sp. SbA4	acidobacteria	A0A2U3KIR3	50°07′N 11°52′E^51−53^
*CabS*TYR	*Caballeronia* sp. SBC1	proteobacteria	A0A6G8NL20	62°57′N 67°03′E^54^
*SinA*TYR	*S. acidiphila* ATCC BAA-1392	planctomycetes	L0D705	58°14′N 38°12′E^55,56^
*PseS*TYR	*Pseudomonas* sp. MWU12–2323	proteobacteria	A0A6I1NHB1	MA, USA^57^
*ChrS*TYR	*Chromobacterium sphagni*	proteobacteria	A0A1S1WVR1	WV and ME, USA^58^

a The respective host organisms and their phylum, UniProt identifiers of the corresponding TYR gene, and geographic location of the habitat are reported. The presence of the respective host organism in a wetland environment has been reported in a previous review published by our research group.^4^

Genes coding for wetland TYRs have been obtained by whole gene synthesis (Eurofins Genomics, Ebersberg, Germany), and the corresponding TYR enzymes (Table 1) have been successfully expressed (as determined by sodium dodecyl-sulfate polyacrylamide gel electrophoresis (SDS-PAGE) and electrospray ionization mass spectrometry (ESI-MS); Figures S3 and S4 and Table S1) using protocols developed and optimized for each of the five TYRs. Optimized parameters included the purification tag, expression time, expression temperature, and lysis buffer composition. Expression conditions as well as expression yields of the respective active and purified enzymes are reported in Table S2. Bacterial TYRs have previously been expressed using a His-tag,^44,59−63^ while several expression protocols of plant and fungal TYRs utilize a GST-tag.^42,64−66^ Both of these tags (His-tag and GST-tag) were successfully employed in this study to produce active enzymes in high yields and purity (Table S2). Expression yields for purified and active TYRs ranged from 4.1 mg/L of expression medium (*CabS*TYR) to 65 mg/L of expression medium (*CanS*TYR). In comparison, the literature-reported expression yields of bacterial TYR enzymes varied between 10 mg/L expression medium (*Streptomyces castaneoglobisporus*)^59^ and 80 mg/L expression medium (*Bacillus megaterium*).^60^*CanS*TYR, *CabS*TYR, and *SinA*TYR showed maximum yields when expressed at low temperatures (10–12 °C, Table S2) and displayed significantly reduced yields (less than 10%) at expression temperatures of 20 °C or higher. Previously reported expression temperatures for bacterial TYRs ranged between 16 °C (*Bacillus aryabhattai*)^61^ and 37 °C (*B. megaterium*).^60^ Although higher expression temperatures allow for shorter expression times, we propose that low-temperature expression provides a suitable way to increase the yield of recombinantly expressed bacterial TYRs.

### Phenolic Compounds in Wetland Ecosystems Act as TYR Substrates

**Figure 2:**
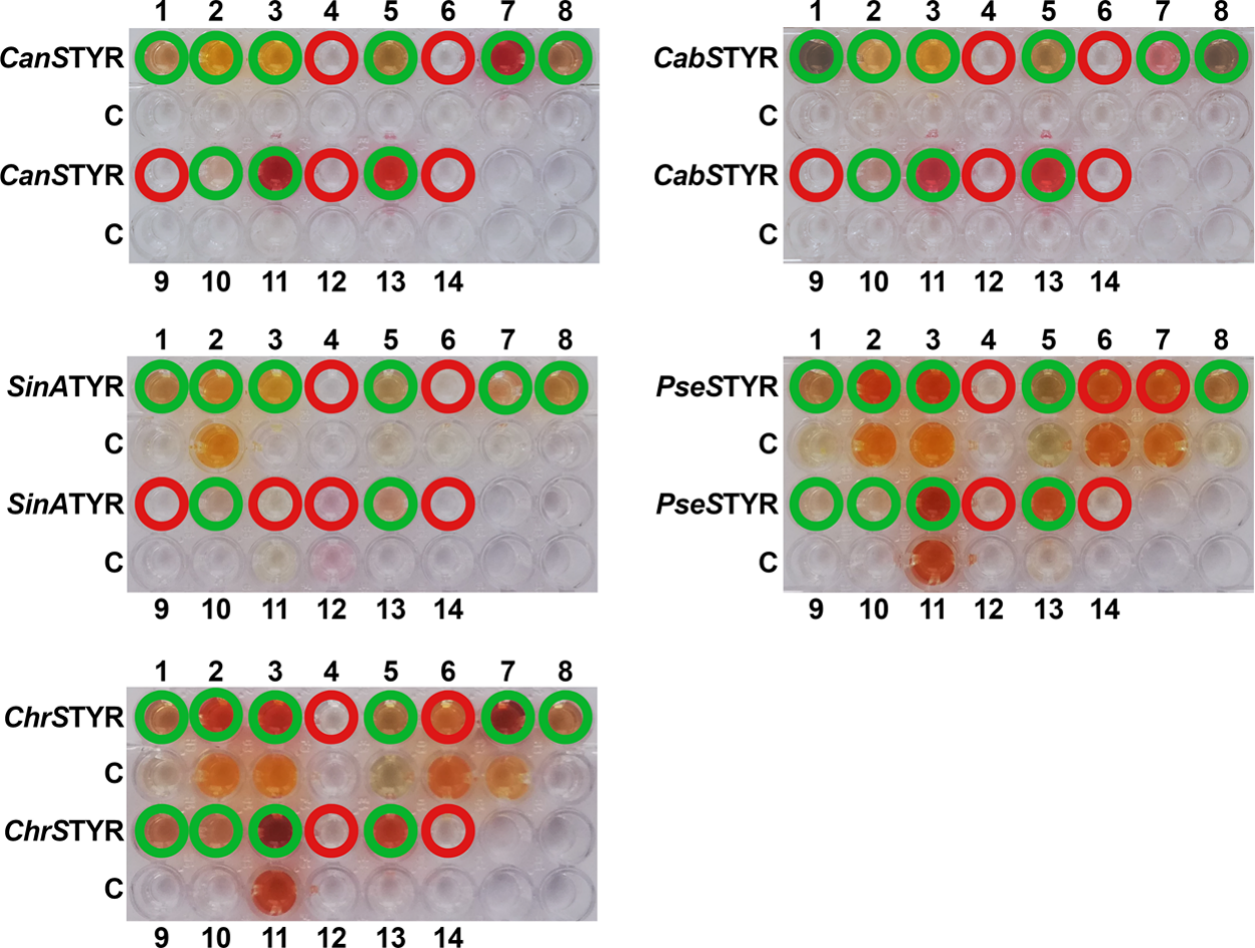
Activity Assays. 1 = caffeic acid, 2 = catechin, 3 = epicatechin, 4 = ferulic acid, 5 = gallic acid, 6 = isorhamnetin, 7 = kaempferol, 8 = *p*-coumaric acid, 9 = *p*-hydroxybenzoic acid, 10 = protocatechuic acid, 11 = quercetin, 12 = syringic acid, 13 = taxifolin, and 14 = vanillic acid. The chemical structures of all substrates are displayed in Figure S1. Active substrate–enzyme combinations are labeled with a green circle, while inactive substrate–enzyme combinations are labeled with a red circle. Rows labeled “C” represent negative controls, which contain the respective low-molecular-weight phenolic compound but no TYR. Changes in color in the control lanes (“C”) are due to the pH-dependent, nonenzymatic autoxidation of the respective substrate. The experimental setup is described in detail in the Materials and Methods Section. The figure has been edited using GIMP 2.10.18 (https://www.gimp.org).

To impact carbon storage in an environmental setting, it is a prerequisite for TYRs to accept phenolic compounds that are naturally abundant within wetland ecosystems. Phenolic compounds previously identified within wetland ecosystems include monophenols (*p*-coumaric acid^34,37^ and *p*-hydroxybenzoic acid^34^), diphenols (protocatechuic acid^34^ and caffeic acid^34,35^), triphenols (gallic acid^34^), methoxylated phenols (ferulic acid,^34,37^ vanillic acid,^34^ and syringic acid^34^), and flavonoids (catechin,^36^ epicatechin,^36^ isorhamnetin,^35^ kaempferol,^35,37^ quercetin,^35,37^ and taxifolin,^36^Figure S1). Substrate scope assays (Figure 2) revealed that a variety of phenolic compounds (including monophenols, diphenols, triphenols, and flavonoids) naturally present within wetland ecosystems are accepted by TYRs as substrates. However, none of the methoxylated compounds (ferulic acid, syringic acid, vanillic acid, and isorhamnetin) are accepted by any of the five investigated TYR enzymes (Figure 2). In accordance, there is no literature report of a TYR enzyme accepting a methoxylated substrate.^4^ Thus, it can be concluded that the rejection of methoxylated phenolic compounds is a general property of TYRs. As a result, methoxylated substrates, although abundant in wetland ecosystems, are not affected by TYR activity.

In contrast, caffeic acid, catechin, epicatechin, gallic acid, *p*-coumaric acid, protocatechuic acid, and taxifolin (Figure S1) were accepted by all investigated TYRs (Figure 2). Thus, wetland ecosystems rich in these compounds can be expected to show an increased response to elevated levels of TYR activity. Kaempferol (accepted by *CanS*TYR, *CabS*TYR, *SinA*TYR, and *ChrS*TYR; rejected by *PseS*TYR), *p*-hydroxybenzoic acid (accepted by *PseS*TYR and *ChrS*TYR; rejected by *CanS*TYR, *CabS*TYR, and *SinA*TYR), and quercetin (accepted by *CanS*TYR, *CabS*TYR, *PseS*TYR, and *ChrS*TYR; rejected by *SinA*TYR) were partially accepted (Figures 2 and S1) and can, therefore, be expected to be involved in the “latch mechanism” to a variable extent dependent on the substrate scope of TYRs present in the respective wetland ecosystem. In a previous study, a TYR from a *Streptomyces* species isolated from globally distributed wetlands (located in Austria^67^ and China^68^) has been demonstrated to accept phenolic compounds naturally present within its habitat. Accordingly, the results presented herein demonstrate that TYRs present within the genomes of phylogenetically diverse and globally distributed host organisms indigenous to wetland ecosystems accept a broad scope of phenolic (nonmethoxylated) compounds present within their natural environment (Figure 2), which substantiates their postulated impact on the wetland carbon cycle.^4,13^

### Kinetic Investigations Reveal Catalytic Preferences of Wetland TYRs

Kinetic parameters (*k*_cat_ and *K*_m_) were determined for recombinantly expressed TYRs (Table 2), which revealed that while all investigated TYR enzymes accept a broad scope of substrates, they show varying substrate preferences (Table 2). In terms of activity (*k*_cat_ values), diphenols are preferred over monophenols, which is in accordance with previous reports.^4,29^ When comparing the *k*_cat_ values of corresponding mono- and diphenols (tyramine–dopamine, l-tyrosine–l-DOPA, *p*-coumaric acid–caffeic acid, *p*-hydroxybenzoic acid–protocatechuic acid; Figure S1), all investigated enzymes showed higher activity levels toward the diphenolic substrate, compared to the corresponding monophenolic substrate (Table 2). This trend is in accordance with literature-reported kinetic investigations of bacterial TYRs.^67,69−72^ In an environmental setting, this translates to a more pronounced involvement of diphenols in the wetland carbon cycle compared to monophenols. Moreover, different reaction mechanisms have been postulated for monophenols and diphenols (Figure 1B) with one striking difference being the amount of oxygen consumed.^27,73^ When diphenols are converted, TYRs consume one molecule of O_2_ and generate two reaction products (two *o*-quinones) per reaction cycle. In contrast, when monophenols are converted, TYRs consume one molecule of O_2_ and generate only one reaction product (one *o*-quinone) per reaction cycle (Figure 1B). Thus, in a wetland environment, where oxygen scarcity imposes the limiting factor on TYR activity, it can be speculated that this will further increase the turnover rate for diphenols compared to monophenols and thus further strengthen the involvement of diphenols over monophenols.

**Table 2:** Kinetic Data of Recombinantly Expressing TYR Enzymes^a^

		*CanS*TYR	*CabS*TYR	*SinA*TYR	*PseS*TYR	*ChrS*TYR
tyramine	*k*_cat_ (s^–1^)	19.3 ± 1.4	6.62 ± 0.62	0.146 ± 0.011	36.2 ± 5.5	8.03 ± 0.51
	*K*_m_ (mM)	0.800 ± 0.167	4.23 ± 1.14	0.989 ± 0.214	2.81 ± 0.93	0.376 ± 0.062
l-tyrosine	*k*_cat_ (s^–1^)	6.22 ± 1.37	7.35 ± 0.79	5.55 ± 0.30	12.9 ± 0.6	7.36 ± 0.70
	*K*_m_ (mM)	3.28 ± 0.91	1.03 ± 0.23	0.236 ± 0.047	0.125 ± 0.014	0.613 ± 0.159
dopamine	*k*_cat_ (s^–1^)	20.2 ± 2.6	9.47 ± 0.71	0.233 ± 0.014	82.8 ± 9.3	89.0 ± 7.5
	*K*_m_ (mM)	0.621 ± 0.150	4.36 ± 0.52	1.27 ± 0.21	0.585 ± 0.126	1.55 ± 0.32
l-DOPA	*k*_cat_ (s^–1^)	64.9 ± 5.4	21.8 ± 3.7	21.9 ± 1.5	34.6 ± 4.4	65.4 ± 2.8
	*K*_m_ (mM)	2.75 ± 0.56	6.19 ± 2.18	0.106 ± 0.028	6.05 ± 1.31	2.84 ± 0.24
caffeic acid	*k*_cat_ (s^–1^)	2.65 ± 0.16	3.66 ± 0.60	0.503 ± 0.035	86.0 ± 15.7	2.72 ± 0.18
	*K*_m_ (mM)	0.770 ± 0.135	1.94 ± 0.46	0.745 ± 0.120	10.3 ± 2.9	0.470 ± 0.112
catechin	*k*_cat_ (s^–1^)	68.4 ± 11.2	0.982 ± 0.116	0.207 ± 0.016	6.48 ± 1.04	11.2 ± 0.807
	*K*_m_ (mM)	2.15 ± 0.52	1.94 ± 0.47	0.835 ± 0.167	0.861 ± 0.332	0.798 ± 0.133
epicatechin	*k*_cat_ (s^–1^)	61.8 ± 7.0	n.d.	1.09 ± 0.12	12.8 ± 0.5	52.2 ± 6.1
	*K*_m_ (mM)	1.48 ± 0.28	n.d.	2.16 ± 0.50	1.04 ± 0.10	0.820 ± 0.181
gallic acid	*k*_cat_ (s^–1^)	n.d.	n.d.	n.d.	33.9 ± 7.3	6.23 ± 0.65
	*K*_m_ (mM)	n.d.	n.d.	n.d.	3.18 ± 1.22	23.9 ± 3.3
*p*-coumaric acid	*k*_cat_ (s^–1^)	0.129 ± 0.016	0.0601 ± 0.0062	0.285 ± 0.019	0.413 ± 0.018	0.306 ± 0.026
	*K*_m_ (mM)	0.229 ± 0.071	0.141 ± 0.032	0.552 ± 0.097	0.0654 ± 0.0092	0.138 ± 0.029
*p*-hydroxy-benzoic acid	*k*_cat_ (s^–1^)	n.d.	n.d.	n.d.	2.30 ± 0.28	3.32 ± 0.16
	*K*_m_ (mM)	n.d.	n.d.	n.d.	0.454 ± 0.163	4.46 ± 0.52
protocatechuic acid	*k*_cat_ (s^–1^)	12.1 ± 1.4	n.d.	n.d.	87.3 ± 12.8	4.00 ± 0.28
	*K*_m_ (mM)	2.49 ± 0.59	n.d.	n.d.	0.812 ± 0.228	1.33 ± 0.23
quercetin	*k*_cat_ (s^–1^)	n.d.	0.0772 ± 0.0093	n.d.	0.554 ± 0.059	0.602 ± 0.073
	*K*_m_ (mM)	n.d.	0.702 ± 0.121	n.d.	0.0125 ± 0.0031	0.124 ± 0.025
taxifolin	*k*_cat_ (s^–1^)	5.79 ± 1.18	4.14 ± 0.42	0.0593 ± 0.0033	1.47 ± 0.08	0.824 ± 0.085
	*K*_m_ (mM)	0.837 ± 0.301	0.483 ± 0.083	0.232 ± 0.029	0.124 ± 0.010	0.186 ± 0.043

a *K*_cat_ and *K*_m_ values of standard substrates (tyramine, l-tyrosine, dopamine, l-DOPA) and active natural substrate–enzyme combinations identified during substrate scope assays are reported ±1 standard deviation. “n.d.” indicates substrate–enzyme combinations that were not determined because the enzyme showed no activity toward the respective substrate, the activity was too low to be determined reliably or the solubility of the substrate was too low to allow for the calculation of kinetic parameters. The chemical structures of all substrates are displayed in Figure S1. Molar extinction coefficients and the amounts of enzyme used per measurement are listed in Tables S3 and S4. The experimental setup is descrietailed description of the expressionbed in detail in the Materials and Methods Section.

Analyzing kinetic data from flavonoid substrates (catechin, epicatechin, quercetin, and taxifolin; Figure S1) revealed that the flavanol substrates (catechin and epicatechin) show higher activity levels (*k*_cat_ values), while quercetin (flavonol) and taxifolin (flavanonol) exhibit higher levels of affinity (*K*_m_ values) (Table 2). Thus, TYRs convert flavanol substrates (catechin and epicatechin; Figure S1) more efficiently (>*K*_m_ values), while at low concentrations (<*K*_m_ values, which are predominate in wetland environments), quercetin and taxifolin are converted more efficiently. This data proves that (besides diphenols and monophenols) flavonoids, which are commonly encountered in wetlands such as mangrove forests,^35−37^ are accepted by a phylogenetically diverse set of TYRs as substrates.

### Inhibitory Effect of TYR Activity on β-Glucosidase

**Figure 3:**
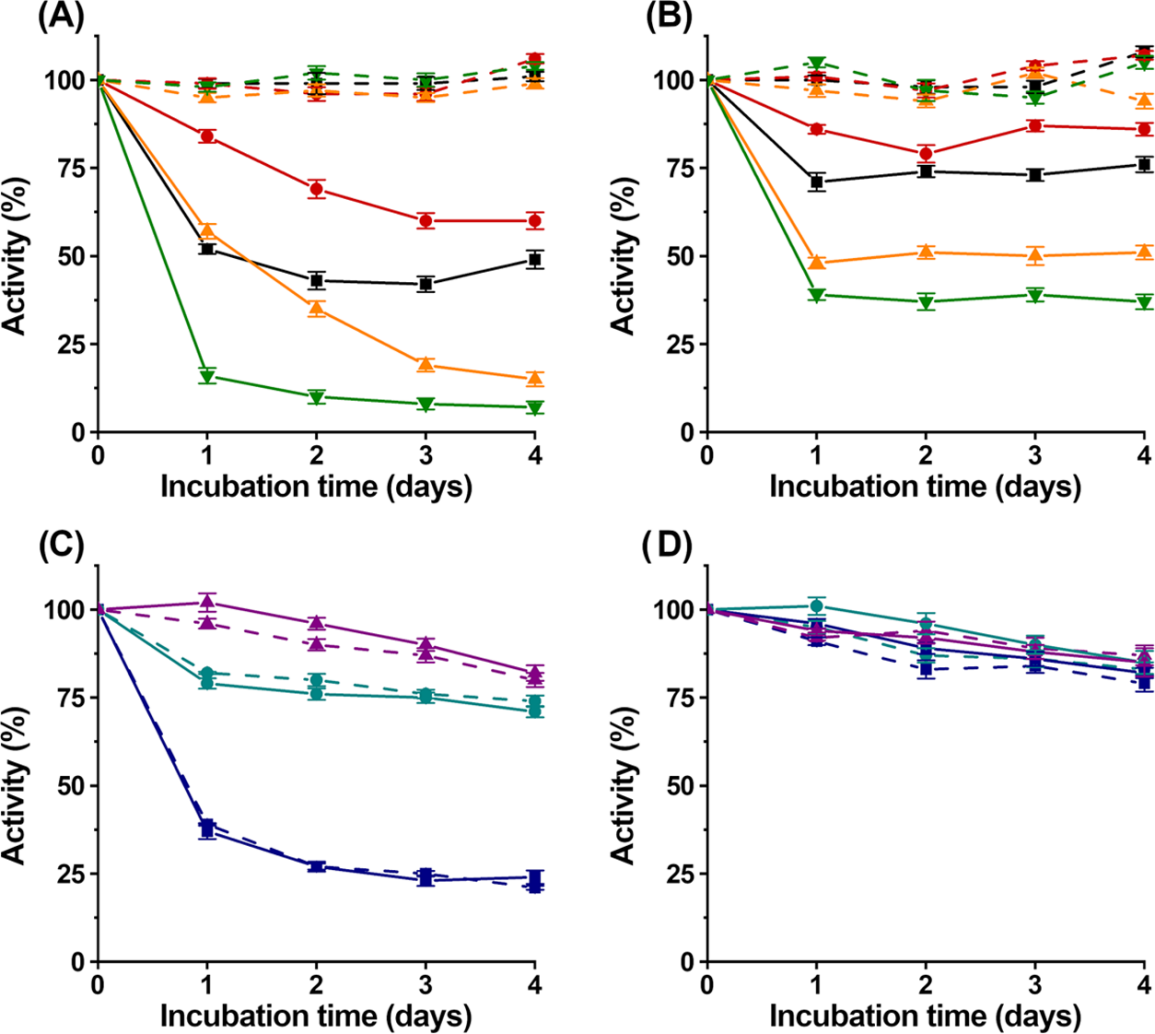
Inhibition of β-glucosidase. (A, B) Inhibition of β-glucosidase by low-molecular-weight phenolic compounds. Black = tyramine, red = catechin, orange = caffeic acid, and green = *p*-coumaric acid (Figure S1). Full lines represent samples supplemented with *SinA*TYR. Dashed lines represent samples without *SinA*TYR. Low-molecular-weight phenolic compounds have been added in concentrations of 1 g/L (A) and 0.1 g/L (B). (C, D) Inhibition of β-glucosidase by high-molecular-weight phenolic compounds. Purple = lignosulfonic acids (52 kDa), cyan = lignosulfonic acids (18 kDa), and blue = humic acids. Full lines represent samples supplemented containing *SinA*TYR (which quantitatively converts phenolic compounds into *o*-quinones, see the Supporting Information). Dashed lines represent samples without *SinA*TYR. High-molecular-weight phenolic polymers have been added at concentrations of 1 (C) and 0.1 g/L (D). Error bars represent one standard deviation (A–D). For a detailed description of the experimental setup, see the Materials and Methods Section. The figure has been created using OriginPro 8 and GIMP 2.10.18 (https://www.gimp.org).

Next, the impact of increased TYR activity on the activities of hydrolytic enzymes was investigated to elucidate the effects of increased TYR activity on the stability of wetland carbon stores. For this purpose, β-glucosidase has been chosen as a model enzyme since its involvement in the hydrolytic decomposition of soil organic matter is well established^13,50,74−76^ and its activity can be determined reliably and accurately using 4-methylumbelliferyl-β-d-glucopyranoside as a substrate.^18,21,77−79^ First, the direct inhibition of β-glucosidase by low-molecular-weight phenolic TYR substrates (tyramine, *p*-coumaric acid, caffeic acid, and catechin; Figure S1) has been investigated. For this purpose, β-glucosidase has been incubated with different concentrations (1 and 0.1 g/L, see the Materials and Methods Section) of low-molecular-weight phenolic compounds (tyramine, catechin, caffeic acid, and *p*-coumaric acid; Figure S1), while β-glucosidase activity has been determined after 24, 48, 72, and 96 h. These measurements revealed only low-level inhibition of β-glucosidase activity by any of the low-molecular-weight phenolic substrates (tyramine, catechin, caffeic acid, and *p*-coumaric acid; Figure 3A,B, dashed lines; Figure S1), compared to β-glucosidase incubated without a low-molecular-weight phenolic compound under the same conditions. This suggests that low-molecular-weight phenolic TYR substrates do not contribute significantly to carbon storage in wetland ecosystems by inhibiting β-glucosidase.

Next, the inhibition of β-glucosidase by low-molecular-weight phenolic compounds in the presence of increased TYR activity has been investigated. For this purpose, β-glucosidase has been mixed with different concentrations (1 and 0.1 g/L, see the Materials and Methods Section) of low-molecular-weight phenolic compounds (tyramine, catechin, caffeic acid, and *p*-coumaric acid; Figure S1). This time, recombinantly expressed *SinA*TYR has been added before incubating the samples (containing β-glucosidase, low-molecular-weight phenolic compounds, and *SinA*TYR) for 4 days with β-glucosidase activity determined after 24, 48, 72, and 96 h (Figure 3A,B, full lines). *SinA*TYR was chosen since it is expressed in an active state and does not require additional SDS activation (Figure S5). Moreover, since *S. acidiphila* is commonly encountered in wetland ecosystems and represents an aerobe organism, efficient expression of *SinA*TYR can be expected in its natural environment, following the aeration of previously anoxic wetland soils due to climate change.^80^ In contrast to our previous experiments, β-glucosidase activity decreased significantly after the addition of *SinA*TYR (compared to the incubation of β-glucosidase with low-molecular-weight phenolic TYR substrates without additional TYR). This inhibition was dependent on the incubation time, the concentration, and the chemical structure of the low-molecular-weight phenolic TYR substrate (Figure 3A,B). For all investigated low-molecular-weight phenolic TYR substrates (tyramine, catechin, caffeic acid, and *p*-coumaric acid; Figure S1), concentration-dependent inhibition has been observed. In the presence of *SinA*TYR, *p*-coumaric acid exhibited the highest β-glucosidase-inhibition potential, followed by caffeic acid, tyramine, and catechin (Figure 3A,B, full lines). The inactivation of β-glucosidase is predominantly caused by quinones (see Supporting Results and Discussion, Table S6), which is a well-documented phenomenon.^81^ It is caused by the reactivity of quinones toward amino groups and thiols (Figure S6), which are commonly present in proteins. This leads to one or more of the following reactions, all of which potentially reduce the enzymatic activity: (a) the direct binding of the quinone to the protein,^81^ (b) the cross-linking of two enzyme molecules,^81^ and (c) the formation of a protein radical.^81^ In the context of TYRs in wetland ecosystems, these results are of significant relevance as they point toward the inactivation of hydrolytic enzymes (such as β-glucosidase) by quinones formed by wetland TYRs following the aeration of previously anoxic wetland soils. Since the reaction products (*o*-quinones) of TYR activity are responsible for this effect and different TYR enzymes generate the identical reaction product from a respective substrate,^27,29^ comparable results can be expected for other TYR enzymes, provided they accept phenolic compounds present in their environment as substrates. This first mechanism might explain investigations reporting decreasing or unchanging emission rates from wetland soils following aeration.^74,82,83^

### Investigations of TYR Activity Toward High-Molecular-Weight Phenolic Polymers

Following investigations focused on the inhibition of β-glucosidase by low-molecular-weight phenolic TYR substrates, the inhibition of hydrolytic enzymes (with β-glucosidase as a model enzyme) by high-molecular-weight phenolic polymers, such as lignin decomposition products (lignosulfonic acid) and humic acids, has been investigated. These compounds have previously been reported to function as suitable representatives for phenolic polymers naturally encountered within wetland ecosystems.^16,76^ First, the inhibition potentials of high-molecular-weight phenolic polymers (humic acids, lignosulfonic acids 18 kDa, lignosulfonic acids 52 kDa) have been determined in the absence of TYR activity, which revealed that humic acids have a more pronounced inhibitory effect on β-glucosidase activity, compared to lignin-derived polymers (Figure 3C,D, dashed lines). These observations are in accordance with previous reports.^76^ Then, β-glucosidase has been incubated with high-molecular-weight phenolic polymers and *SinA*TYR has been added (Figure 3C,D, full lines). Activity measurements revealed that the addition of *SinA*TYR had no significant effect on the inhibition potential of humic acids and lignosulfonic acids. We speculate that this is due to the reception of high-molecular-weight phenolic polymers (such as humic acids and lignosulfonic acids) by TYRs as substrates. To substantiate this hypothesis, molecular docking studies have been performed, which revealed for all five investigated TYRs that high-molecular-weight phenolic polymers, in fact, cannot access the active center of TYRs (Figure S7). In TYRs, the hydroxy groups of low-molecular-weight phenolic substrates are oriented toward the dicopper center in a distance of 2–3 Å (Figure S1),^40,41,84−86^ which is a prerequisite for catalytic activity. In contrast, our docking experiments revealed for all five investigated TYRs that hydroxy groups in high-molecular-weight phenolic polymers are sterically blocked from accessing the active center due to the bulky structure of the polymer (Figure S7). Thus, while high-molecular-weight phenolic polymers have a significant impact on the activities of hydrolytic enzymes, they will not be directly affected by increased TYR activity caused by the aeration of previously anoxic wetland soils.

### Removal of High-Molecular-Weight Phenolic Polymers by Reactive Quinones Generated by TYRs

**Figure 4:**
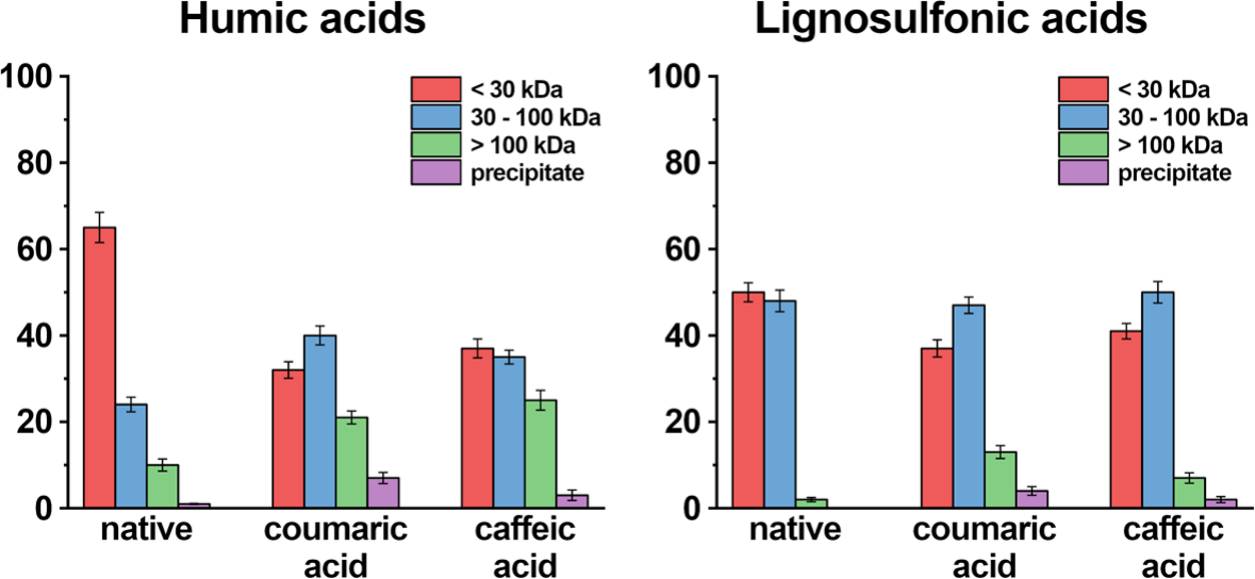
Molecular weight distribution profiles. Humic acids (1 g/L) and lignosulfonic acids (52 kDa, 1 g/L) have been incubated with *p*-coumaric acid (100 mg/L) and caffeic acid (100 mg/L) for 48 h with 1 mg/L *SinA*TYR at pH 7. The “native” sample contains the respective phenolic polymer (1 g/L) and *p*-coumaric acid (100 mg/L) without *SinA*TYR. Error bars represent ±1 standard deviation. Measurements were performed in triplicate. The Figure has been created using OriginPro 8 and GIMP 2.10.18 (https://www.gimp.org).

Finally, the interplay between low-molecular-weight phenolic TYR substrates and high-molecular-weight phenolic polymers (lignosulfonic acids and humic acids) has been investigated for the first time. In a natural wetland environment, low-molecular-weight phenolic compounds are present alongside high-molecular-weight phenolic polymers. Consequently, quinones produced by TYRs (from low-molecular-weight phenolic TYR substrates) can not only react with proteins, as has been demonstrated for β-glucosidase within this study, but also react with high-molecular-weight phenolic polymers. To yield further information on the effects of increased TYR activity on carbon cycling within wetland ecosystems, the interplay between low-molecular-weight phenolic compounds (which are accepted by TYRs as substrates) and high-molecular-weight phenolic polymers (which are inert toward the direct oxidation by TYRs, see above) in the presence of TYR activity has been investigated. Therefore, humic acids and lignosulfonic acids (52 kDa), both of which represent complex mixtures of polymers, have been characterized in terms of their molecular weight distribution profile by fractionation *via* ultrafiltration. This method has previously been shown to yield fractions with similar physicochemical properties (such as elemental composition and the presence of functional groups and chemical bonds), which are separate according to their degree of polymerization.^87^ For humic acids and lignosulfonic acids, the relative quantities (in terms of mass) have been determined for the fraction <30 kDa (humic acids: 66%; lignosulfonic acids: 50%; Figure 4), the fraction ranging from 30–100 kDa (humic acids: 23.5%; lignosulfonic acids: 48%; Figure 4), and for the fraction >100 kDa (humic acids: 10.5%; lignosulfonic acids: 2%; Figure 4). Next, humic acids and lignosulfonic acids (1 g/L each; see the Materials and Methods Section) have been incubated with low-molecular-weight phenolic TYR substrates (*p*-coumaric acid and caffeic acid, 100 mg/L each) and *SinA*TYR (1 mg/L). As a negative control, humic acids and lignosulfonic acids (1 g/L each) have been incubated with *p*-coumaric acid (100 mg/L) but without *SinA*TYR. The molecular weight distribution profiles of humic acids and lignosulfonic acids have been redetermined after 48 h and revealed substantial shifts in their degree of polymerization for samples with added *SinA*TYR (Figure 4). In contrast, after incubation for 48 h, control samples showed no shifts in the degree of polymerization (Figure S8). For humic acids and lignosulfonic acids, the molecular weight distribution shifted toward higher molecular weights, indicating an increased degree of polymerization. This effect has been observed to a similar degree for the monophenolic substrate *p*-coumaric acids and for the corresponding diphenolic substrate caffeic acid (Figure S1) and has been more pronounced for humic acids, compared to lignosulfonic acids (Figure 4). Interestingly, a fraction amounting to 3–7% (w/w) for humic acids and 2–4% (w/w) for lignosulfonic acids has been removed from the solution by precipitation (Figure 4). The available data suggest that these effects are caused by *o*-quinones. The precise structure of *o*-quinones generated by TYRs is dependent on the respective structure of the corresponding substrate but independent of the architecture of the TYR enzyme.^27,29^ Thus, similar results can be expected for TYR enzymes, in general.

*o*-quinones can interact with high-molecular-weight phenolic polymers in two main ways. They can either bind directly to the polymer, thus adding their molecular weight to the mass of the polymer, or they perform redox exchange. *o*-quinones have been shown to react with thiol groups, the sulfur atom of thioether groups, amino groups, and phenolic hydroxy groups, and, therefore, *o*-quinones have the potential to react with humic acids and lignosulfonic acids (Figure S6).^88^ On the other hand, redox exchange leads to the reduction of a quinone (produced from a low-molecular-weight phenolic compound by a TYR) to the corresponding diphenol, while the redox-exchange-partner (*e.g.*, a diphenolic group in the high-molecular-weight phenolic polymer) gets oxidized to the corresponding quinone (Figure S6).^88^ This “secondary quinone” (formed *via* redox exchange) can then undergo the aforementioned reactions, which can consequently lead to the cross-linking of two polymer molecules, which drastically increases the molecular mass of the resulting molecule. Since the solubility of humic substances is negatively correlated to their molecular weight,^89,90^ this process can result in the precipitation of the cross-linked polymer, as has been observed in our study (Figure 4). Precipitation of phenolic polymers may lead to reduced inhibition of hydrolytic enzymes, which will potentially stimulate organic matter degradation following the aeration of previously anoxic wetland soils. However, further investigations are required to determine the extent and stability of precipitated phenolic polymers.

## Materials and Methods

### Sequence Selection, Expression, and Purification of Recombinantly Expressed TYRs

Five TYR sequences previously identified within the genomes of bacteria indigenous to wetland ecosystems^4^ were selected to cover different bacterial phyla (Acidobacteria, Proteobacteria, Planctomycetes) and to originate from different geographic locations (Table 1).

The codon-optimized (toward the codon usage of *Escherichia coli*) nucleotide sequences coding for *CanS*TYR, *CabS*TYR, *SinA*TYR, *PseS*TYR, and *ChrS*TYR (Table S1) were obtained from a commercial supplier (Eurofins Genomics, Ebersberg, Germany) and cloned into the pGEX-6P-SG expression vector^91^ adjacent to either a GST-tag (*CanS*TYR, *PseS*TYR, and *ChrS*TYR) or a 10xHis-tag (*CabS*TYR and *SinA*TYR) using the restriction endonuclease *Esp*3I *via* restriction enzyme recognition sites introduced into the optimized sequence. The ORFs were sequence verified in the forward and reverse directions using Sanger sequencing provided by a commercial supplier (Microsynth GmbH, Vienna, Austria).

All enzymes were heterologously expressed in *E. coli* BL21(DE3) and purified using affinity chromatography (GST-tag: *CanS*TYR, *PseS*TYR, and *ChrS*TYR or 10xHis-tag: *CabS*TYR and *SinA*TYR). The successful expression of TYR enzymes was checked using SDS-PAGE (Figure S3) and ESI-MS (Figure S4 and Table S1). A detailed description of the expression and purification procedure can be found in the Supporting Materials and Methods Section.

### Determination of the Substrate Scope

The determination of the SDS optima and pH optima of recombinantly expressed TYRs is described in detail in the Supporting Materials and Methods Section. 100 μg of recombinantly expressed and purified TYRs was mixed with 1 mM of one of the following phenolic substrates: caffeic acid, catechin, dopamine, epicatechin, ferulic acid, gallic acid, l-DOPA, *p*-coumaric acid, *p*-hydroxybenzoic acid, protocatechuic acid, syringic acid, tyramine, l-tyrosine, and vanillic acid (Figure S1). The activities toward the flavonoid substrates isorhamnetin, kaempferol, quercetin, and taxifolin, which do not yield colored reaction products and show poor solubility in water, were assessed in a reaction mixture containing 10% DMSO supplemented with 5 mM MBTH (3-methyl-2-benzothiazolinone hydrazine hydrochloride hydrate). Optimal SDS molarities (Figure S5 and Table S5) were added, and the pH of the reaction mixture was adjusted with 50 mM MES (2-(*N*-morpholino) ethanesulfonic acid; *CanS*TYR and *CabS*TYR) or 50 mM Tris (tris(hydroxymethyl)aminomethane; *SinA*TYR, *PseS*TYR, and *ChrS*TYR) to the pH optimum of the respective enzyme (Figure S9 and Table S5). The reactions were performed in a total volume of 200 μL. The activity was assessed visually by a clear change in color within 24 h at 22 °C.

### Kinetic Investigations

Kinetic parameters (Table 2) for enzyme–substrate combinations exhibiting activity were determined photometrically by measuring the maximum reaction rate of the formation of the colored reaction products on a TECAN infinite M200 reader. Absorption wavelengths and the corresponding molar absorption coefficients have either been reported previously^64,92^ or have been determined within the scope of this study (Table S3 and Figure S10).^64,92^ Adequate amounts of the TYR enzyme (Table S4) were mixed with a substrate in a 50 mM buffer solution (MES buffer: *CanS*TYR and *CabS*TYR; Tris buffer: *SinA*TYR, *PseS*TYR, and *ChrS*TYR; adjusted to the pH optimum of the respective TYR enzyme, Table S5 and Figure S9) supplemented with previously identified optimal SDS molarities (Table S5 and Figure S5). Maximum reaction rates were determined for seven to eight substrate molarities per substrate–enzyme combination in triplicate. Nonlinear curve fitting of the Michaelis–Menten equation toward the measured reaction rates implemented in OriginPro 8 software was used to calculate *K*_m_ values and *k*_cat_ values (Figures S11–S15).

### Inhibition of β-Glucosidase

β-glucosidase (1 mg/L, Sigma-Aldrich, Vienna, Austria) was incubated with tyramine, *p*-coumaric acid, caffeic acid, catechin, lignosulfonic acids (18 and 52 kDa), and humic acids (1 and 0.1 g/L, respectively; all compounds were purchased from Sigma-Aldrich) in 50 mM Tris-HCl (pH 7.0). *SinA*TYR (1 mg/L) was added to the mixture for samples with TYR activity. Samples solutions were incubated at 4 °C and β-glucosidase activity has been determined after 24, 48, 72, and 96 h.

4-Methylumbelliferyl-β-d-glucopyranoside (Sigma-Aldrich) was used to measure β-glucosidase activity. Fluorescence measurements were performed on a TECAN infinite M200 reader (Tecan, Salzburg, Austria, excitation wavelength: 365 nm, emission wavelength: 455 nm)^77^ in triplicate. For each measurement, 10 μL of sample solution was mixed with 5 μL of 10 mM 4-methylumbelliferyl-β-d-glucopyranoside (dissolved in 100% 2-methoxyethanol). The solution was filled up to 200 μL with H_2_O and adjusted to 50 mM Tris-HCl (pH 7.0). As a control sample, β-glucosidase (1 mg/L) was incubated at 4 °C in 50 mM Tris-HCl (pH 7.0) without additional phenolics.

### Determination of the Molecular Weight Distribution Profiles of Phenolic Polymers

Vivaspin ultrafiltration devices (MWCO 30 kDa, VWR) and Microsept advance ultrafiltration devices (MWCO 100 kDa, VWR) were used for the fractionation of phenolic polymers. Phenolic polymers were separated by centrifuging at 4.000 g at 20 °C. Phenolic contents in the supernatant (Vivaspin: >30 kDa, Microsept: >100 kDa) and flow-through (Vivaspin: <30 kDa, Microsept: <100 kDa) were determined gravimetrically in triplicate after removing the solvent (water) *via* evaporation.

## Supporting Information Available

Results and Discussion, Materials and Methods; UniProt identifiers, molar extinction coefficients, and molecular masses (calculated and measured) of recombinantly expressed TYR enzymes; expression and purification of recombinantly expressed TYRs; affinity values for docking poses of humic acid; chemical structures of low-molecular-weight phenolic compounds; SDS profiles of recombinantly expressed TYRs; docking of a humic acid to the active sites of wetland TYRs; and nonlinear curve fitting for active substrates with CanSTYR (PDF)
