# Supplementary material for: Biochemical Investigations of Five Recombinantly Expressed Tyrosinases Reveal Two Novel Mechanisms Impacting Carbon Storage in Wetland Ecosystems
Source: Environ Sci Technol. 2023 Sep 1;57(37):13863–73. doi: 10.1021/acs.est.3c02910 (PMC10515480; doi:10.1021/acs.est.3c02910)
Supplement: Supplementary file 1 — es3c02910_si_001.pdf [file es3c02910_si_001.pdf]

Supporting Information.

Biochemical investigations of five recombinantly expressed tyrosinases reveal two novel mechanisms impacting carbon storage in wetland ecosystems.

Felix Panis [1], Annette Rompel *[1]

1 Universität Wien, Fakultät für Chemie, Institut für Biophysikalische Chemie, Josef-Holaubek-Platz 2, 1090 Wien, Austria; https://www.bpc.univie.ac.at/en/

*Correspondence to: annette.rompel@univie.ac.at

Summary Information:

Number of Pages: 35

Number of Tables: 7

Number of Figures: 15

Table of contents

Supplementary Results and Discussions……S3

Inhibition of β-glucosidase by polymerization products……S3

o-quinone production in incubation samples……S3

Supplementary Materials and Methods……S4

Expression and purification of *Can*STYR, *Cab*STYR, *Sin*ATYR, *Pse*STYR, *Chr*STYR……S4

Molecular mass determination……S5

Determination of the pH optima and the SDS optima of recombinantly expressed *Can*STYR, *Cab*STYR, *Sin*ATYR, *Pse*STYR, *Chr*STYR……S5

Molecular docking……S5

Inhibition of β-glucosidase by polymerization products……S6

o-quinone production in incubation samples……S6

Supplementary Tables……S8

Table S1. UniProt identifiers, molar extinction coefficients, and molecular masses (calculated and measured) of recombinantly expressed TYR enzymes……S8

Table S2. Expression and purification of recombinantly expressed TYRs……………………………………..S8
Table S3. Wavelengths and absorption coefficients used to determine kinetic parameters………..S9
Table S4: Amounts of TYR enzyme used for kinetic measurements…………………………………………S10
Table S5. pH optima and SDS optima of *CanS*TYR, *CabS*TYR, *SinA*TYR, *PseS*TYR, *ChrS*TYR……………S10
Table S6. Inhibition of β-glucosidase by polymerization products………………………………………….S11
Table S7. Affinity values for docking poses of the humic acid shown in Figure S7………………………S13
Supplementary Figures………………………………………………………………………………………………………..S14
Figure S1. Chemical structures of low-molecular-weight phenolic compounds…………………………..S15
Figure S2. Active center of TYRs…………………………………………………………………………………………S16
Figure S3. Reducing SDS-PAGE of recombinantly expressed TYRs……………………………………………S16
Figure S4. Positive mode ESI-LTQ-Orbitrap Velos mass spectra of *CanS*TYR, *CabS*TYR, *SinA*TYR, *PseS*TYR, *ChrS*TYR………………………………………………………………………………………………………S17
Figure S5. SDS profiles of recombinantly expressed TYRs………………………………………………………S18
Figure S6. Chemical reactivities of *o*-quinones……………………………………………………………………S19
Figure S7. Docking of a humic acid to the active sites of wetland TYRs……………………………………S21
Figure S8. Molecular weight distribution profiles of negative controls……………………………………S21
Figure S9. pH profiles of recombinantly expressed TYRs………………………………………………………..S22
Figure S10. Determination of molar extinction coefficients……………………………………………………S23
Figure S11. Non-linear curve fitting for active substrates with *CanS*TYR…………………………………S25
Figure S12. Non-linear curve fitting for active substrates with *CabS*TYR…………………………………S27
Figure S13. Non-linear curve fitting for active substrates with *SinA*TYR…………………………………S29
Figure S14. Non-linear curve fitting for active substrates with *PseS*TYR…………………………………S31
Figure S15. Non-linear curve fitting for active substrates with *ChrS*TYR…………………………………S33
References……S34

Supplementary Results and Discussions.

Inhibition of β-glucosidase by polymerization products. Since polymerization products are formed spontaneously by *o*-quinones (originating from *SinA*TYR activity)[1,2] in incubation samples containing β-glucosidase in combination with low-molecular-weight phenolic compounds (tyramine, catechin, *p*-coumaric acid, and caffeic acid) and *SinA*TYR, their relative inhibitory effect on β-glucosidase, compared to *o*-quinones, has been evaluated (see Supplementary Materials and Methods). This revealed that polymerization products formed by *SinA*TYR from phenolic precursors (tyramine, catechin, *p*-coumaric acid, and caffeic acid) play a secondary role in the inhibition of β-glucosidase (6 – 14 %, Table S6), compared to *o*-quinones produced *in-situ* by *SinA*TYR (86 – 94 %, Table S6).

***o*-quinone production in incubation samples.** The extent of the conversion of phenolic substrates into *o*-quinones by *SinA*TYR in incubation samples containing β-glucosidase in combination with low-molecular-weight phenolic compounds (tyramine, catechin, *p*-coumaric acid, and caffeic acid) and *SinA*TYR has been evaluated. For this purpose, measurements of the *o*-quinone production rates have been performed and revealed that no new quinones are produced after incubation at 4 °C for 96 hours. However, when additional phenolic substrate (tyramine, 1 mM) was added to the incubation mixture the formation of new *o*-quinones could be detected. Thus, it can be concluded that the non-production of *o*-quinones in samples incubated for 96 hours (without additional phenolic substrates) is caused by the quantitative conversion of phenols into the corresponding *o*-quinones.

Supplementary Materials and Methods.

Expression and purification of *CanS*TYR, *CabS*TYR, *SinA*TYR, *PseS*TYR, *ChrS*TYR. For the recombinant expression of TYR enzymes (Table S1), the sequence-verified expression vector was transformed into chemically competent *E. coli* BL21(DE3) cells, which were subsequently used to inoculate 500 ml of autoclaved LB medium (10 g/l NaCl, 10 g/l tryptone, 5 g/l yeast extract) containing 100 µg/ml ampicillin. The expression batches were incubated at 37 °C and 230 rpm until an OD_{600} value of 0.8−1.0 was reached. Then, 0.5 mM isopropyl-β-D-thiogalactopyranoside (IPTG) and 0.5 mM $CuSO_4$ were added. The expression time and the expression temperature were optimized individually for all recombinantly expressed TYRs and are listed in Table S2. After 72 – 96 h (Table S2), the expression medium was centrifuged for 10 min at 4000 g at 4 °C, and the cell pellets were resuspended in 50 ml of a cell lysis buffer. The composition of the lysis buffer has been optimized for each of the TYRs individually (Table S2). The cell lysis was performed by three repetitive cycles of freezing the samples in liquid nitrogen, followed by thawing in a water bath (20 °C). The highly viscous cell lysate was incubated after the addition of 0.03 g/l DNase I (Sigma-Aldrich, Vienna, Austria) and 7 mM $MgCl_2$ at 4 °C under shaking for 10 minutes. Then, the samples were centrifuged at 20.000 g for 15 minutes and filtered before they were applied to either a 5 ml GSTrap FF column (GE Healthcare, Freiburg, Germany; *CanS*TYR, *PseS*TYR, and *ChrS*TYR) or to a HisTrap Excel 1 ml column (GE Healthcare, Freiburg, Germany; *CabS*TYR and *SinA*TYR) *via* an ÄKTA explorer system placed in a refrigerator at 4 °C. Samples were washed on the column using pure cell lysis buffer (Table S2) and eluted using cell lysis buffer supplemented with either 10 mM reduced glutathione (*CanS*TYR, *PseS*TYR, and *ChrS*TYR) or 500 mM imidazole (*CabS*TYR and *SinA*TYR).

For *CanS*TYR, *PseS*TYR, and *ChrS*TYR, the purified GST-TYR fusion protein was buffer exchanged to a glutathione-free cell lysis buffer (Table S2) using Vivaspin ultrafiltration devices (MWCO 30 kDa, VWR, cumulative dilution: 1:10.000). Next, a GST-tagged HRV-3C-protease[3] was added at a mass ratio of 1:75 (protease:TYR) and the mixture was placed at 4 °C for 24 hours. The TYR enzyme was purified by applying the mixture to a 5 ml GSTrap FF column. While the GST-tag and the GST-tagged-protease bound to the column, the TYR enzyme passed through the column and was collected.

*CabS*TYR and *SinA*TYR were purified using a HisTrap Excel 1 ml column. After elution of the 10xHis tagged protein using the respective lysis buffer supplemented with 500 mM imidazole, the buffer was exchanged to an imidazole-free cell lysis buffer (Table S2).

The purified proteins (*CanS*TYR, *CabS*TYR, *SinA*TYR, *PseS*TYR, *ChrS*TYR) were quantified using the Lambert-Beer law by measuring the absorption of the protein at 280 nm applying

extinction coefficients calculated by the ExPASy ProtParam tool (Table S1)[4]. The purity of the proteins was determined by a reducing SDS-PAGE (12.5%, Figure S3). All enzymes were stored in the respective cell lysis buffer at 4 °C and were used immediately for further investigations.

Molecular mass determination. The samples were first loaded on a trap column µ-Precolumn 5 mm x 300 µm i.d. C4 PepMap300, 5 µm, 300 Å (Thermo Scientific) with 0.1% trifluoroacetic acid. Separation was carried out on a C4 analytical column 50 cm x 75 µm Accucore C4, 2.6 µm, 150 Å (Thermo Fisher Scientific) at a flow rate of 300 nl/min. Mobile phase A: (2% acetonitrile, 98% H_2O, 0.1% formamide) Mobile phase B: (80% acetonitrile, 20% H_2O, 0.1% formamide). Mass spectra were obtained using an LTQ Orbitrap Velos mass spectrometer (Thermo Fisher Scientific, Bremen, Germany) equipped with a nanospray ion source. The electrospray voltage was 2.1 kV and the ion transfer capillary temperature was 300 °C. Full MS scans were acquired in positive ion mode in the 400-2000 m/z range at a resolution of 7500 (FWHM at 400m/z).

Determination of the pH optima and the SDS optima of recombinantly expressed *CanS*TYR, *CabS*TYR, *SinA*TYR, *PseS*TYR, *ChrS*TYR. For the determination of the pH optima, 1 M buffer stock solution (pH 2 – 7.0: NaOH – citric acid buffer, pH 7.0 – 9.0: TRIS – HCl, pH 9.0 – 11.0: CAPS – HCl buffer) was added to a final concentration of 50 mM to each TYR enzyme (Table S4), mixed with 1 mM tyramine (Figure S1, P) and 2 mM SDS in a total volume of 200 µl. The pH optima were determined in increments of 0.5 pH units by measuring the maximum reaction rate of the formation of the colored reaction product (dopaminochrome) at 480 nm (Table S3 & S4, Figure S9)[5]. All measurements were performed on a TECAN infinite M200 reader (Tecan, Salzburg, Austria).

For the determination of the SDS optima different SDS molarities (0 – 20 mM) were added to the TYR enzymes, mixed with 1 mM tyramine and 50 mM buffer solution (adjusted to the pH value that resulted in maximum activity for the respective TYR enzyme) in a total volume of 200 µl. All measurements were performed on a TECAN infinite M200 reader (Tecan, Salzburg, Austria) by measuring the maximum reaction rate of the formation of the colored reaction product (dopaminochrome) at 480 nm (Table S3 & S4, Figure S5)[5].

Molecular docking. Molecular docking was performed using AutoDock Vina[6]. The structures of *CanS*TYR, *CabS*TYR, *SinA*TYR, *PseS*TYR, and *ChrS*TYR were obtained using

AlphaFold[7,8]. pdbqt files of the enzymes were created using AutoDockTools (ADT, v. 1.5.6) with added Gasteiger charges[6]. 20 poses were calculated for each target and substrate with the exhaustiveness set to 100. The substrate (humic acid) was designed according to a literature source[9]. All rotatable bonds have been defined as rotatable. The docking settings (i.e. the grid box) were tested using *CanS*TYR, *CabS*TYR, *SinA*TYR, *PseS*TYR, and *ChrS*TYR and *L*-tyrosine, which yielded docking poses for all enzymes similar to the orientation of *L*-tyrosine in the crystal structure of the TYR from *Bacillus megaterium* (*Bm*TYR) (Figure S2). Binding poses were searched in a 30 x 30 x 30 Å grid box enclosing the two copper ions of the active site. Humic acid docking poses in which a hydroxyl oxygen atom (of the humic acid) was positioned within 1 Å of the hydroxylic oxygen atom in the docking pose of tyrosine in the structure from *Bm*TYR (Figure S2) were evaluated as reasonable while other docking poses were evaluated as unreasonable. If no reasonable docking pose was found the pose with the highest affinity has been selected.

Inhibition of β-glucosidase by polymerization products. Incubation samples contained *SinA*TYR (1 mg/l), one of the low-molecular-weight phenolic compounds (tyramine, catechin, *p*-coumaric acid, and caffeic acid, 1 g/l and 0.1 g/l, each) and β-glucosidase (1 mg/l) in 50 mM TRIS – HCl (pH 7.0). A control sample was prepared containing *SinA*TYR (1 mg/l) and β-glucosidase (1 mg/l) in 50 mM TRIS – HCl (pH 7.0) without phenolic compounds. After incubation for 96 hours at 4 °C, the samples were heated to 95 °C for 15 minutes. The samples were cooled to 4 °C and β-glucosidase (1 mg/l) was added. Again, the samples were incubated for 96 hours at 4 °C. β-glucosidase activity was determined for all samples as described in the main manuscript (please see: Materials and Methods “Inhibition of β-glucosidase”). The level of inhibition (%) has been calculated for incubation samples relative to the control sample.

***o*-quinone production in incubation samples.** The level of *in-situ o*-quinone production after incubation at 4 °C for 96 hours was determined for incubation samples containing β-glucosidase (1 mg/l) in combination with one of the low-molecular-weight phenolic compounds (tyramine, catechin, *p*-coumaric acid, and caffeic acid; 1 g/l and 0.1 g/l, respectively) and *SinA*TYR (1 mg/l) incubated at 4 °C for 96 hours by mixing 10 µl of the incubation sample with 50 mM TRIS – HCl (pH 7.0) in a total volume of 200 µl.

To check if active *SinA*TYR is present in incubation samples after incubating at 4 °C for 96 hours, 10 µl of the incubation samples containing β-glucosidase (1 mg/l) in combination with low-molecular-weight phenolic compounds (tyramine, catechin, *p*-coumaric acid, and caffeic

acid; 1 g/l and 0.1 g/l each) and *SinA*TYR (1 mg/l) incubated at 4 °C for 96 hours were mixed with 50 mM TRIS – HCl (pH 7.0) and 1 mM tyramine in a total volume of 200 µl.

o-quinone production rates were measured photometrically in triplicates on a TECAN infinite M200 reader using absorption wavelengths and the corresponding molar absorption coefficients are reported in Table S3.

Supplementary Tables.

Table S1. UniProt identifiers, molar extinction coefficients, and molecular masses (calculated and measured) of recombinantly expressed TYR enzymes. Molar extinction coefficients and molecular masses have been calculated using the ExPASy ProtParam tool (https://web.expasy.org/protparam/). Molecular masses have been determined using ESI-MS (see Materials and Methods in the section "Molecular mass determination", Figure S4).

Enzyme	UniProt IDs	extinction coefficients at 280 nm $(g/l)^{-1}$	Calculated mass (g/mol)	Molecular mass determined by ESI-MS (g/mol)
*CanS*TYR	A0A2U3KIR3	1.875	57873.5	57873.8
*CabS*TYR	A0A6G8NL20	1.702	53310.9	53310.0
*SinA*TYR	L0D705	2.070	40650.0	40649.4
*PseS*TYR	A0A6I1NHB1	1.979	54163.9	54164.5
*ChrS*TYR	A0A1S1WVR1	1.465	55752.1	55752.8

Table S2. Expression and purification of recombinantly expressed TYRs. The UniProt IDs, the expression times, the expression temperatures, the cell lysis buffers used for resuspending the cell pellet, for performing the freeze-thaw-cycles, and for chromatography, the purification tag attached to the respective TYR sequence, and the yield of pure and active TYR after the purification process are listed (given in mg protein per liter of expression culture).

Enzyme	UniProt ID	Expression time	Expression temperature	Cell lysis buffer	Purification tag	Yield (mg/l)
*CanS*TYR	A0A2U3KIR3	4 days	12 °C	5 mM EDTA<br>50 mM NaCl<br>50 mM Tris<br>pH 9.0	GST-tag	65.0
*CabS*TYR	A0A6G8NL20	4 days	10 °C	75 mM NaCl<br>10 mM Imidazole<br>50 mM Tris<br>pH 9.0	10xHis-tag	4.1
*SinA*TYR	L0D705	4 days	10 °C	50 mM NaCl<br>10 mM Imidazole<br>50 mM Tris<br>pH 9.0	10xHis-tag	61.3
*PseS*TYR	A0A6I1NHB1	3 days	25 °C	5 mM EDTA<br>300 mM NaCl<br>50 mM Tris<br>pH 7.0	GST-tag	20.2
*ChrS*TYR	A0A1S1WVR1	3 days	20 °C	100 mM NaCl<br>10 mM Imidazole<br>50 mM Tris<br>pH 9.0	GST-tag	25.3

Table S3. Wavelengths and absorption coefficients used to determine kinetic parameters. Wavelengths and absorption coefficients have either been reported previously in the literature or have been determined within the scope of this study (Figure S10). The final volume was 200 µl for all of these measurements. The chemical structures of all substrates are displayed in Figure S1.

**substrate**	**λ_{max} (nm)**	**$\varepsilon_{\lambda max}$ ($l\ mol^{-1}\ cm^{-1}$)**
tyramine	480[5]	3300[5]
L-tyrosine	475[5]	3600[5]
dopamine	480[5]	3300[5]
L-DOPA	475[5]	3600[5]
protocatechuic acid	454[10]	213[10]
gallic acid	346[10]	4140[10]
p-coumaric acid	495[5]	2062[5]
caffeic acid	495[5]	2062[5]
quercetin	522[10]	31825[10]
taxifolin	516[10]	35325[10]
p-hydroxybenzoic acid	454[10]	213[10]
catechin	377 (this work)	4120 (this work)
epicatechin	371 (this work)	5530 (this work)

Table S4: Amounts of TYR enzyme used for kinetic measurements. Amounts of TYR enzyme in µg used for kinetic measurements for the determination of the pH optima, the SDS optima, and kinetic parameters of phenolic compounds naturally present within wetland ecosystems. Chemical structures of all substrates are displayed in Figure S1. "n.d." labels inactive substrate – enzyme combinations for which kinetic parameters could not be determined.

**substrate**	***CanS*TYR**	***CabS*TYR**	***SinA*TYR**	***PseS*TYR**	***ChrS*TYR**
pH optimum	4.02 µg	0.870 µg	25.1 µg	1.15 µg	2.57 µg
SDS optimum	4.02 µg	0.870 µg	25.1 µg	1.15 µg	2.57 µg
caffeic acid	5.55 µg	3.60 µg	9.80 µg	0.529 µg	18.8 µg
catechin	1.37 µg	3.60 µg	7.50 µg	2.27 µg	6.28 µg
dopamine	3.88 µg	1.80 µg	19.0 µg	0.227 µg	0.523 µg
epicatechin	1.33 µg	2.00 µg	10.7 µg	0.908 µg	0.628 µg
ferulic acid	n.d.	n.d.	n.d.	n.d.	n.d.
gallic acid	n.d.	n.d.	n.d.	0.529 µg	23.0 µg
isorhamnetin	n.d.	n.d.	n.d.	n.d.	n.d.
kaempferol	n.d.	n.d.	n.d.	n.d.	n.d.
L-DOPA	1.94 µg	1.08 µg	0.280 µg	0.561 µg	1.05 µg
p-coumaric acid	23.0 µg	100 µg	10.7 µg	15.1 µg	23.6 µg
p-hydroxybenzoic acid	n.d.	n.d.	n.d.	22.7 µg	47.1 µg
protocatechuic acid	39.7 µg	n.d.	n.d.	3.79 µg	23.6 µg
quercetin	15.5 µg	28.0 µg	n.d.	3.03 µg	1.04 µg
syringic acid	n.d.	n.d.	n.d.	n.d.	n.d.
taxifolin	2.20 µg	0.565 µg	5.35 µg	0.379 µg	0.251 µg
tyramine	5.82 µg	1.17 µg	19.0 µg	2.271 µg	1.57 µg
L-tyrosine	13.6 µg	0.780 µg	0.580 µg	1.14 µg	1.57 µg
vanillic acid	n.d.	n.d.	n.d.	n.d.	n.d.

Table S5. pH optima and SDS optima of *CanS*TYR, *CabS*TYR, *SinA*TYR, *PseS*TYR, *ChrS*TYR. A detailed description of the experiment is provided in the Supplementary Materials and Methods section in the subsection "Determination of the pH optima and the SDS optima of recombinantly expressed *CanS*TYR, *CabS*TYR, *SinA*TYR, *PseS*TYR, *ChrS*TYR".

**Enzyme**	**pH optimum**	**SDS optimum (mM)**
*CanS*TYR	pH 6.0	2.0
*CabS*TYR	pH 6.5	9.0
*SinA*TYR	pH 7.0	1.5
*PseS*TYR	pH 8.0	5.0
*ChrS*TYR	pH 8.0	4.0

Table S6. Inhibition of β-glucosidase by polymerization products. The inhibition of β-glucosidase by polymerization products spontaneously formed by *o*-quinones has been investigated. *o*-quinones are generated *in-situ* by *Sin*ATYR from tyramine, catechin, *p*-coumaric acid, and caffeic acid (1 g/l and 0.1 g/l, respectively). “Inhibitory effect (%) of polymerization products” reports the level of β-glucosidase inhibition, compared to a control sample free of polymerization products. **“**Inhibitory effect (%) of polymerization products and *o*-quinones” reports the level of β-glucosidase inhibition, compared to a control sample free of *o*-quinones and polymerization products. “Relative inhibitory effect (%) of *o*-quinones” reports the inhibitory effect of *o*-quinones, compared to the inhibitory effect of *o*-quinones and polymerization products. “Relative inhibitory effect (%) of polymerization products” reports the inhibitory effect of *o*-quinones, compared to the inhibitory effect of *o*-quinones and polymerization products. For a detailed description of the experimental setup see Supplementary Materials and Methods.

**Substrate**	**Inhibitory effect (%) of polymerization products**	**Inhibitory effect (%) of polymerization products and *o*-quinones**	**Relative inhibitory effect (%) of *o*-quinones**	**Relative inhibitory effect (%) of polymerization products**
**1 g/l**				
tyramine	51.5±2.1	4.92±0.20	9.56±0.55	90.4±0.6
catechin	43.4±1.7	3.48±0.13	8.02±0.43	92.0±0.4
caffeic acid	84.9±3.2	10.4±0.4	12.3±0.6	87.7±0.6
p-coumaric acid	93.3±2.9	5.44±0.28	5.83±0.35	94.2±0.4
**0.1 g/l**				
tyramine	29.6±1.8	3.98±0.16	13.4±1.0	86.6±1.0
catechin	19.6±1.7	2.02±0.12	10.3±1.1	89.7±1.1
caffeic acid	45.7±1.5	6.49±0.14	14.2±0.6	85.8±0.6
p-coumaric acid	64.8±3.3	4.88±0.09	7.54±0.40	92.5±0.4

Table S7: Affinity values for docking poses of the humic acid shown in Figure S7. For a detailed description of the docking process see Materials and Methods in the section "Molecular docking".

Enzyme	Affinity (kcal/mol)
*CanS*TYR	-10.9
*CabS*TYR	-11.3
*SinA*TYR	-11.1
*PseS*TYR	-11.0
*ChrS*TYR	-11.8

Supplementary Figures.

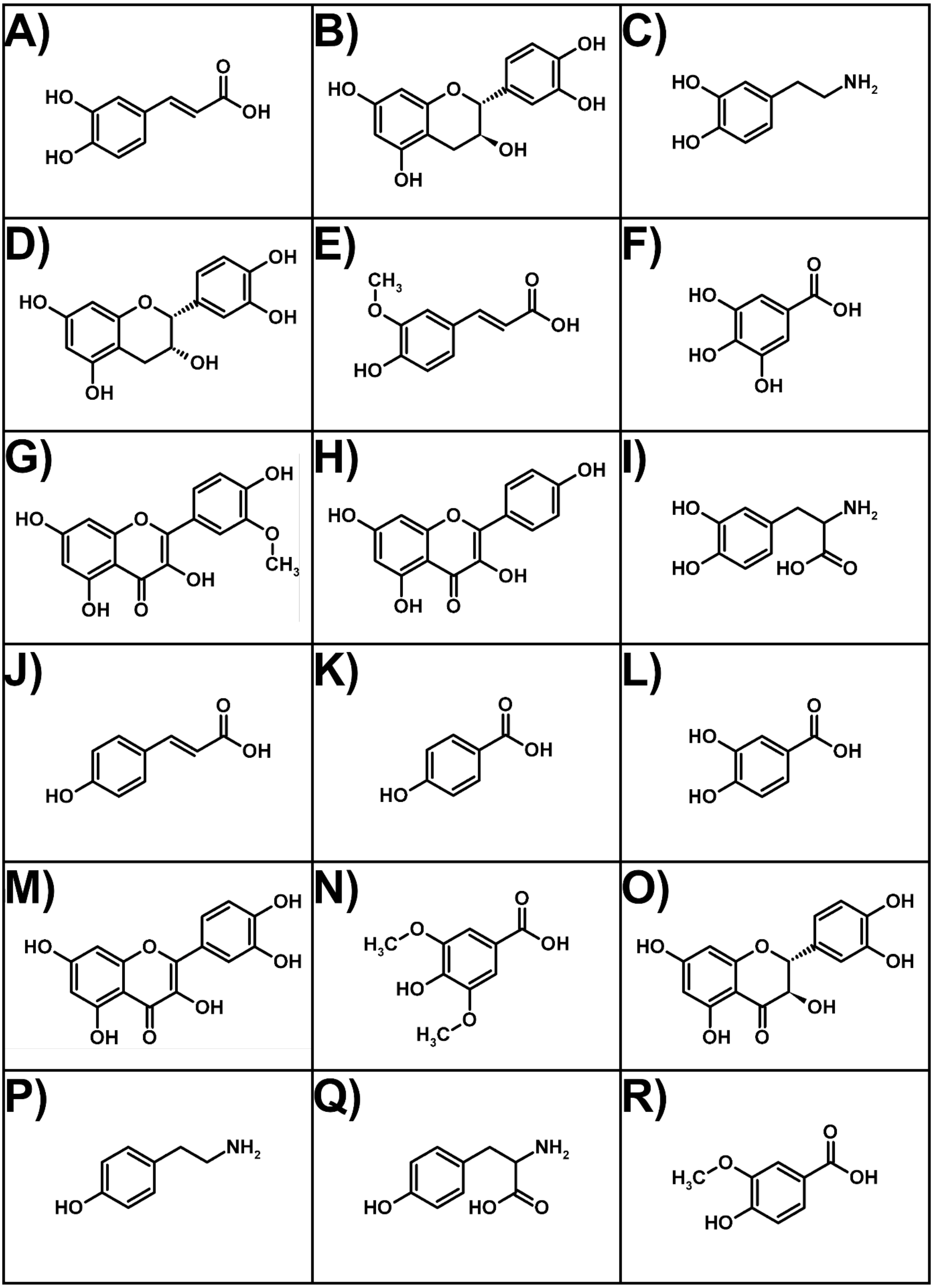

S)

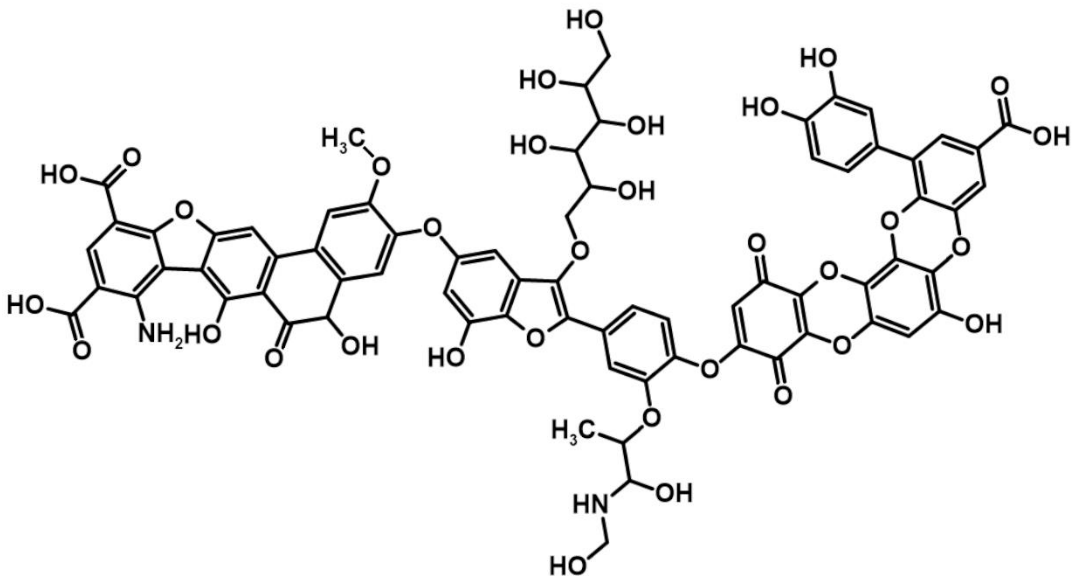

Figure S1. Chemical structures of low-molecular-weight phenolic compounds. A = caffeic acid, B = catechin, C = dopamine, D = epicatechin, E = ferulic acid, F = gallic acid, G = isorhamnetin, H = kaempferol, I = *L*-DOPA, J = *p*-coumaric acid, K = *p*-hydroxybenzoic acid, L = protocatechuic acid, M = quercetin, N = syringic acid, O = taxifolin, P = tyramine, Q = *L*-tyrosine, R = vanillic acid, S = humic acid used for docking experiments.

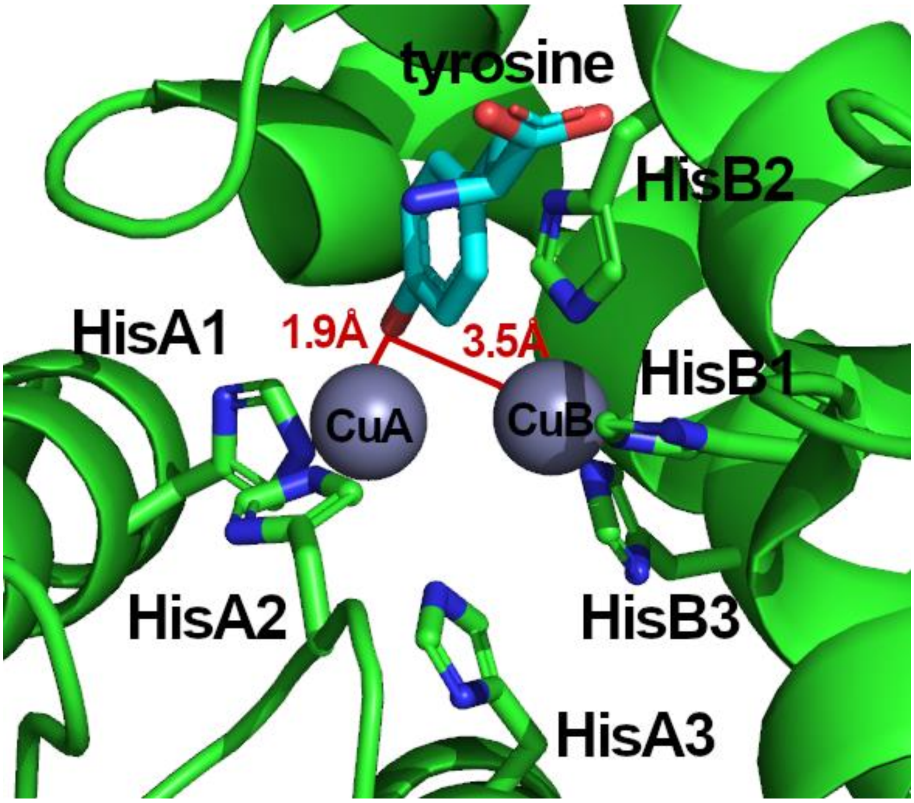

Figure S2. Active center of TYRs. The crystal structure from *Bacillus megaterium* crystallized with *L*-tyrosine in the active site based on PDB: 4P6R[11]. The two copper ions (CuA and CuB) are coordinated by three conserved histidine residues, each (CuA: HisA1, HisA2, HisA3; CuB: HisB1, HisB2, HisB3). *L*-tyrosine is located 1.9 Å from CuA and 3.5 Å from CuB.

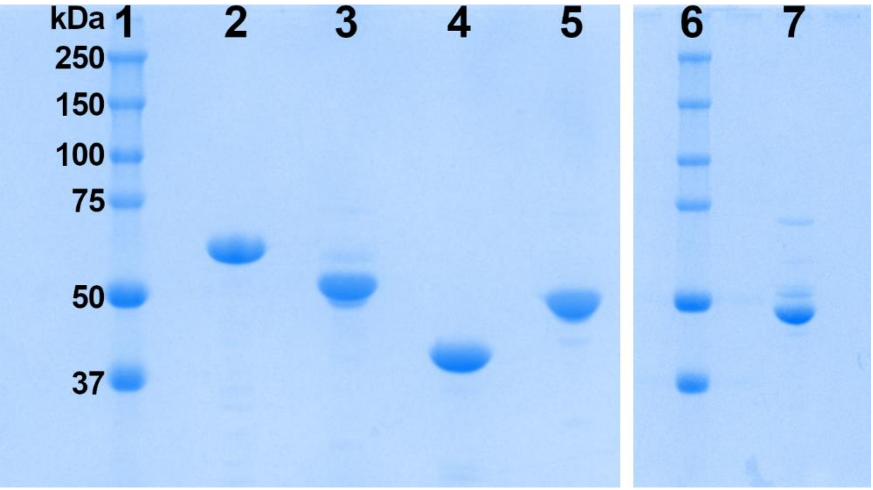

Figure S3. Reducing SDS-PAGE of recombinantly expressed TYRs. 1 = marker, 2 = *CanS*TYR, 3 = *CabS*TYR, 4 = *SinA*TYR, 5 = *PseS*TYR, 6 = marker, 7 = *ChrS*TYR. The calculated masses of the recombinantly expressed enzymes are listed in Table S1. Molecular weights have additionally been verified using an LTQ Orbitrap Velos mass spectrometer (see Materials and Methods in the section "Molecular mass determination" and Figure S4).

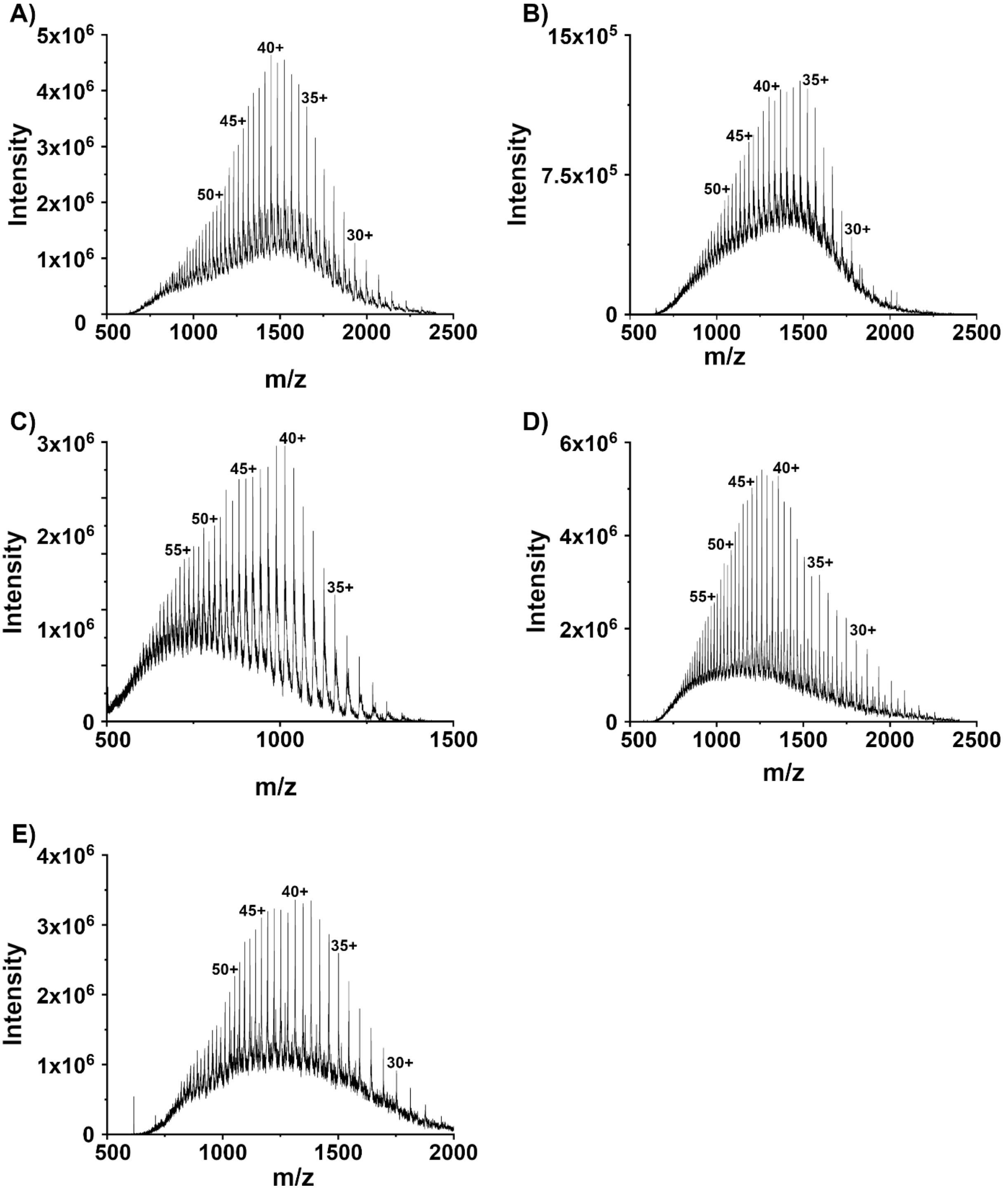

Figure S4. Positive mode ESI-LTQ-Orbitrap Velos mass spectra of *CanS*TYR, *CabS*TYR, *SinA*TYR, *PseS*TYR, *ChrS*TYR. The calculated and measured masses of the proteins are listed in Table S1. The peak labels correspond to the charge state (z) of the respective peaks. A = *CanS*TYR, B = *CabS*TYR, C = *SinA*TYR, D = *PseS*TYR, E = *ChrS*TYR. Detailed information about the experimental setup is provided in the Materials and Methods in the subsection “Molecular mass determination”.

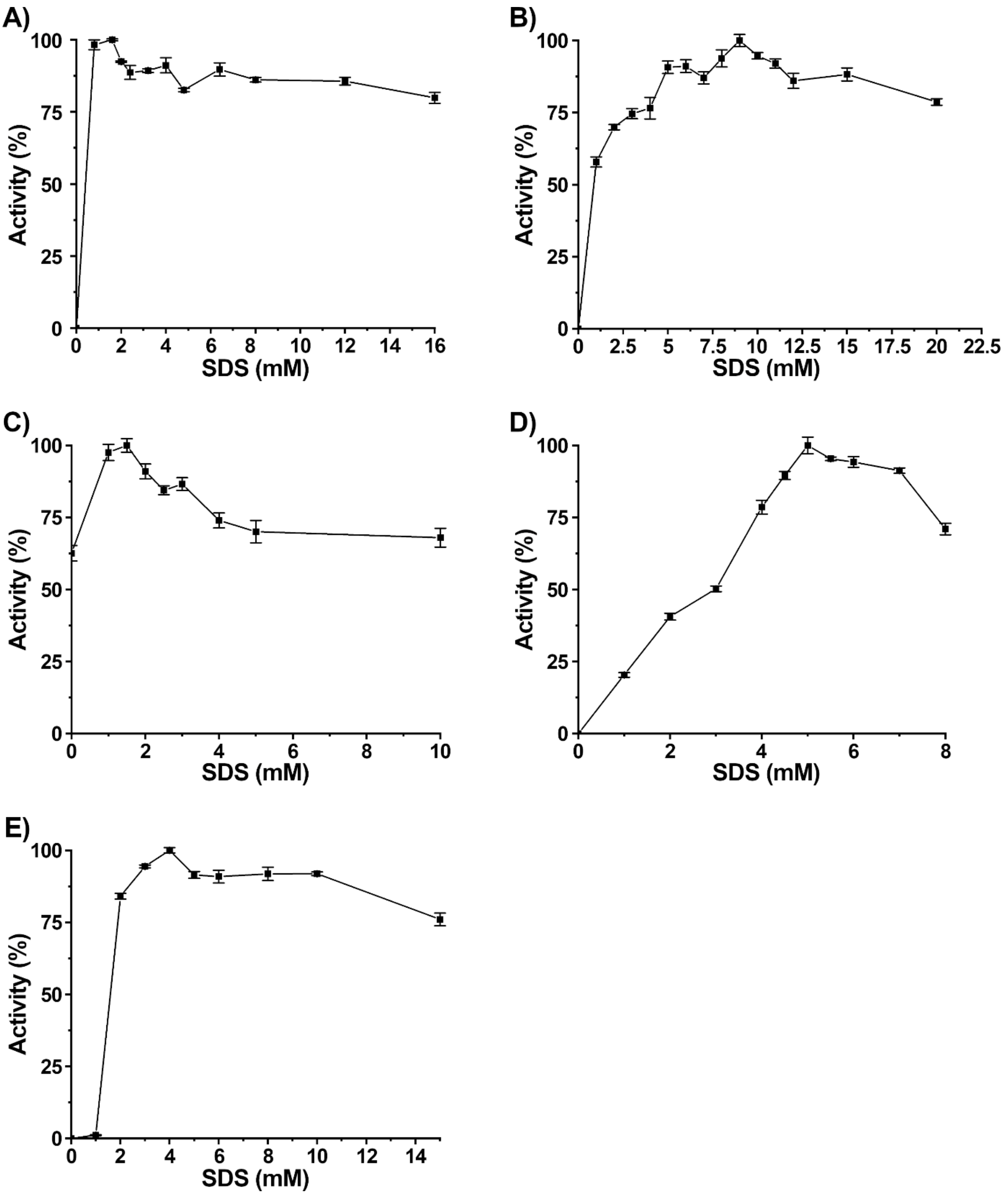

Figure S5. SDS profiles of recombinantly expressed TYRs. A = *Can*STYR, B = *Cab*STYR, C = *Sin*ATYR, D = *Pse*STYR, and E = *Chr*STYR. Measurements were performed in triplicate on a TECAN infinite M200 reader using tyramine (Figure S1, P) as the substrate (1 mM). The error bars indicate ±1 standard deviation. Detailed information on the experimental setup is provided in the Supplementary Materials and Methods section. The SDS optima are listed in Table S5. Activation of TYRs by SDS has been reported to be a suitable method to overcome the enzymes latency *in-vitro*[12–14]. *In-vivo*, activation of latent TYRs has been reported to be

achieved by an autolytic self-cleavage reaction[15]. The Figure has been created using OriginPro 8 and GIMP 2.10.18 (https://www.gimp.org).

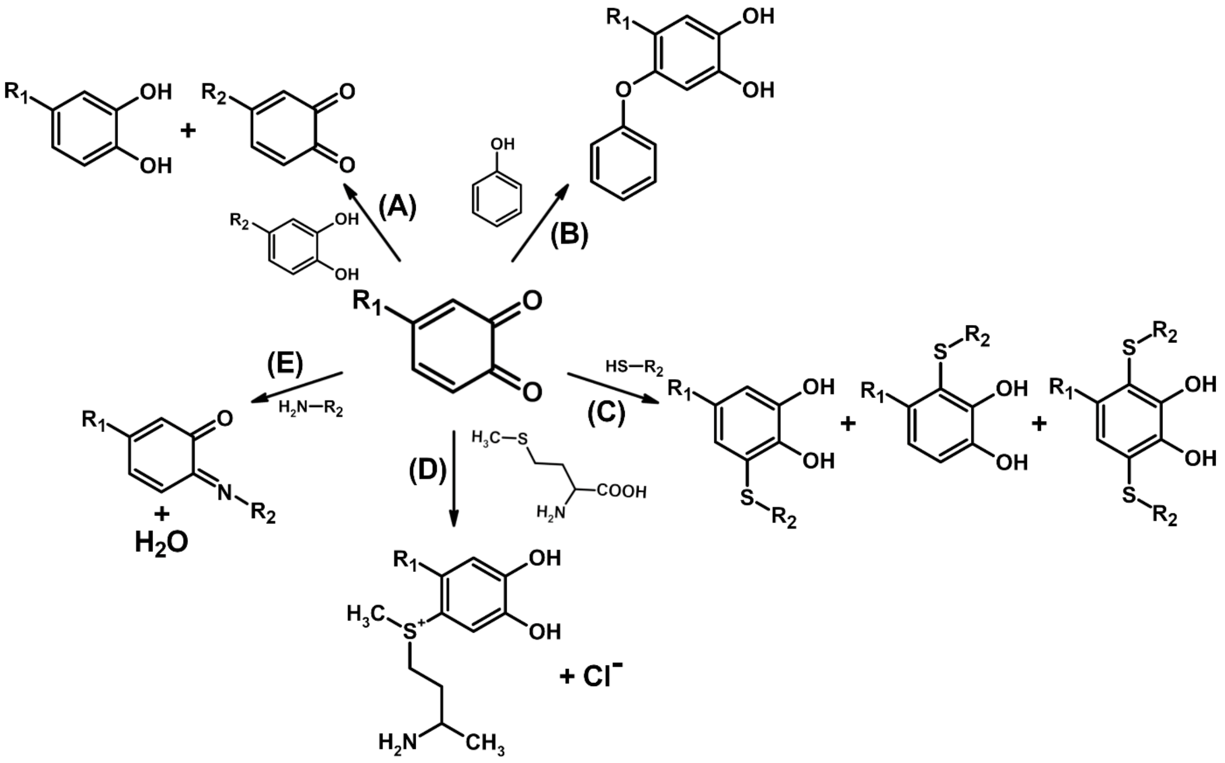

Figure S6. Chemical reactivities of *o*-quinones.[16] **A**: redox exchange with a diphenol leads to the reduction of a quinone to the corresponding diphenol while the redox-exchange partner gets oxidized to the corresponding quinone. **B**: reaction of a quinone with a phenolic hydroxy group. **C**: reaction of a quinone with a thiol group. **D**: reaction of a quinone with a thioether group of a methionine. **E**: reaction of a quinone with a primary amine.

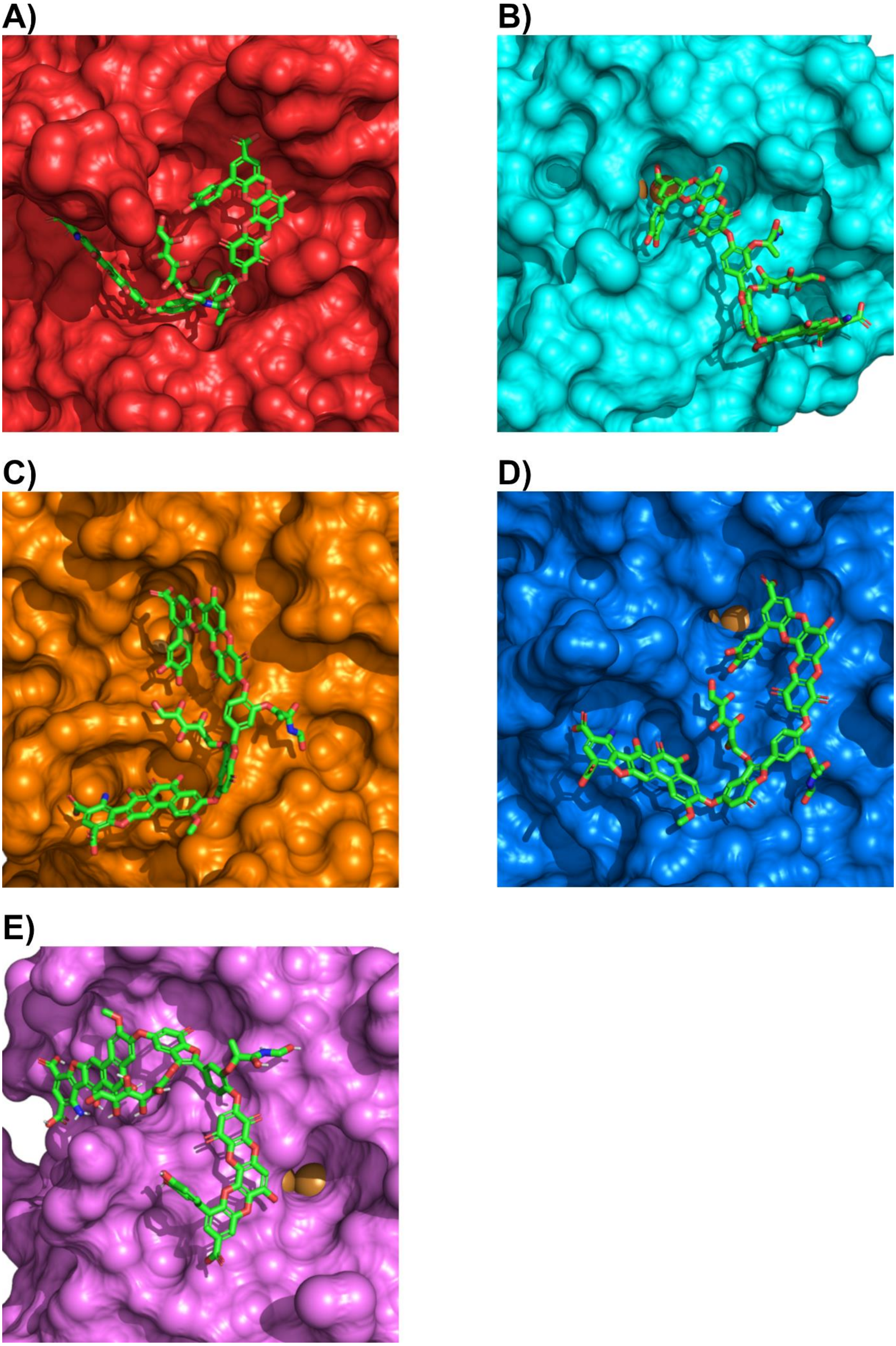

Figure S7. Docking of a humic acid to the active sites of wetland TYRs. A = *Can*STYR, B = *Cab*STYR, C = *Sin*ATYR, D = *Pse*STYR, E = *Chr*STYR. Due to the bulky structure of high-molecular-weight phenolic polymers, they cannot access the active center of TYRs in the correct orientation. The humic acid has been designed according to literature reports[9,17–19] (Figure S1, S). Affinity values for the respective docking poses are shown in Table S7. Detailed information on the experimental setup is given in the Materials and Methods section. The Figure has been created using PyMOL 2.5 (https://pymol.org/2/) and GIMP 2.10.18 (https://www.gimp.org).

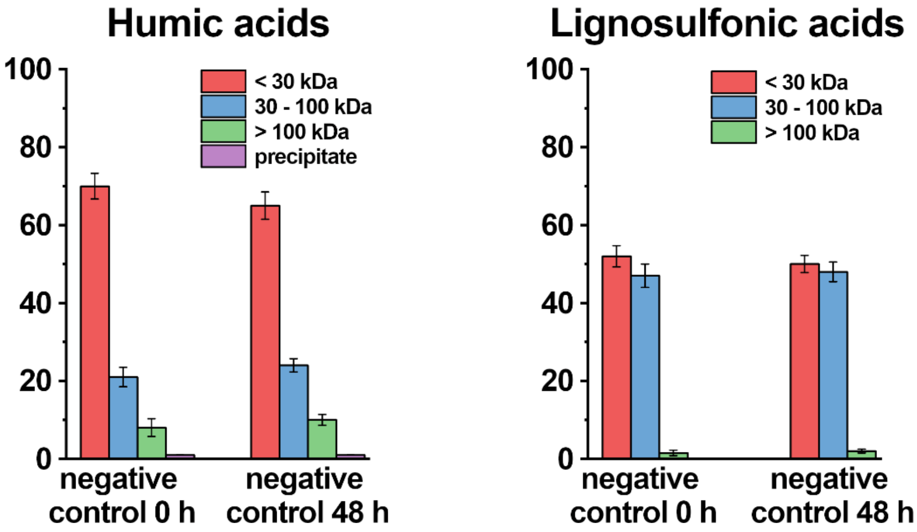

Figure S8. Molecular weight distribution profiles of negative controls. Humic acids (1 g/l) and lignosulfonic acids (52 kDa, 1 g/l) have been incubated with *p*-coumaric acid (100 mg/l) for 48 hours at pH 7 without *Sin*ATYR. Molecular weight distribution profiles for humic acids and lignosulfonic acids have been determined after 0 hours and after 48 hours and, in the absence of *Sin*ATYR (and, consequently, in the absence of *o*-quinones), revealed no significant shifts. Error bars represent ±1 standard deviation. Measurements have been performed in triplicates. The Figure has been created using OriginPro 8 and GIMP 2.10.18 (https://www.gimp.org).

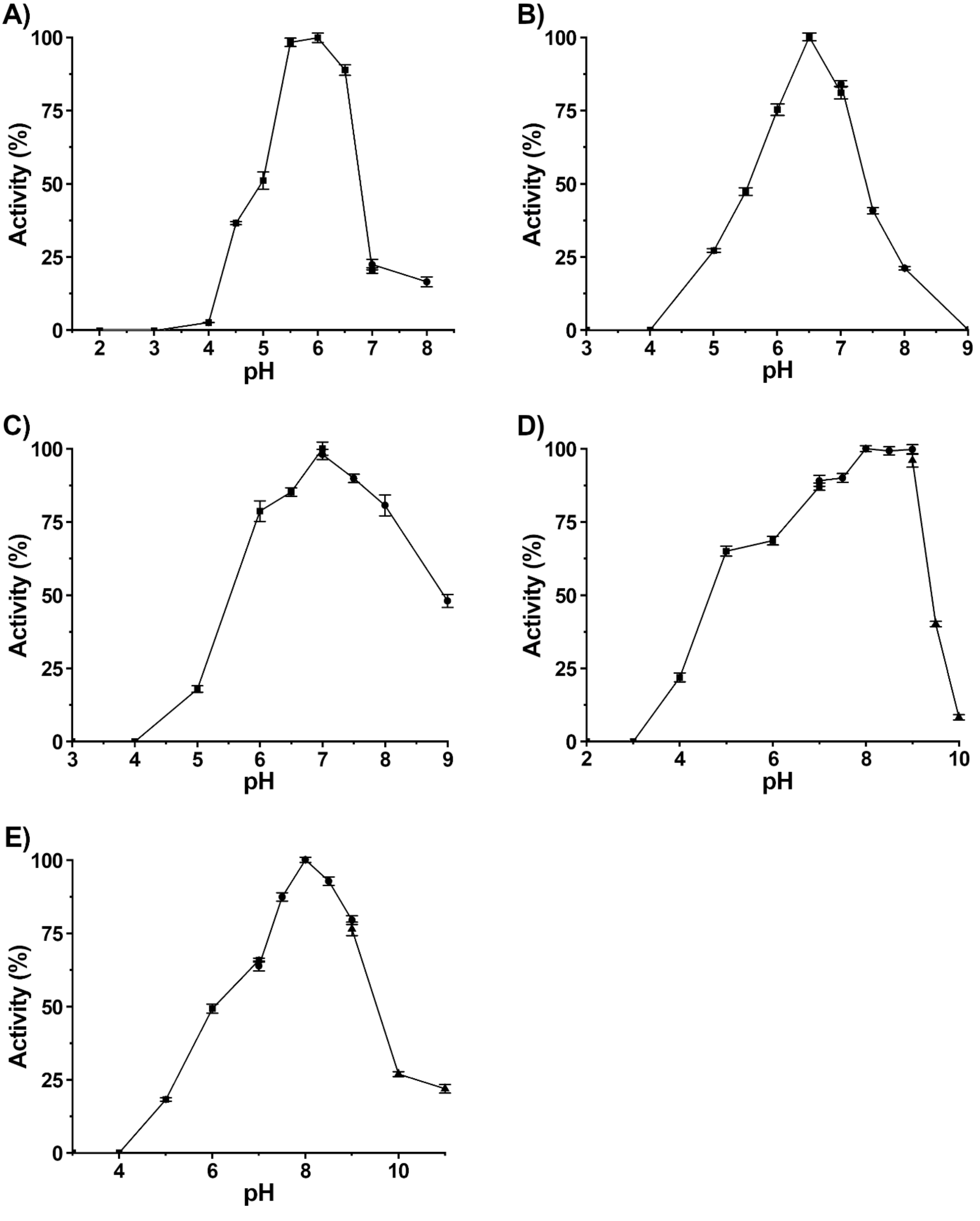

Figure S9. pH profiles of recombinantly expressed TYRs. A = *CanS*TYR, B = *CabS*TYR, C = *SinA*TYR, D = *PseS*TYR, and E = *ChrS*TYR. Measurements were performed in triplicate on a TECAN infinite M200 reader using tyramine as a substrate. The error bars indicate ±1 standard deviation. NaOH – citric acid buffer (pH 2 – 7.0, squares), TRIS – HCl buffer (pH 7.0 – 9.0, circles), and CAPS – HCl buffer (pH 9.0 – 11.0, triangle) have been used. Detailed information on the experimental setup is provided in the Supplementary Materials and Methods

section. pH optima are listed in Table S5 in the Supporting Information. The Figure has been created using OriginPro 8 and GIMP 2.10.18 (https://www.gimp.org).

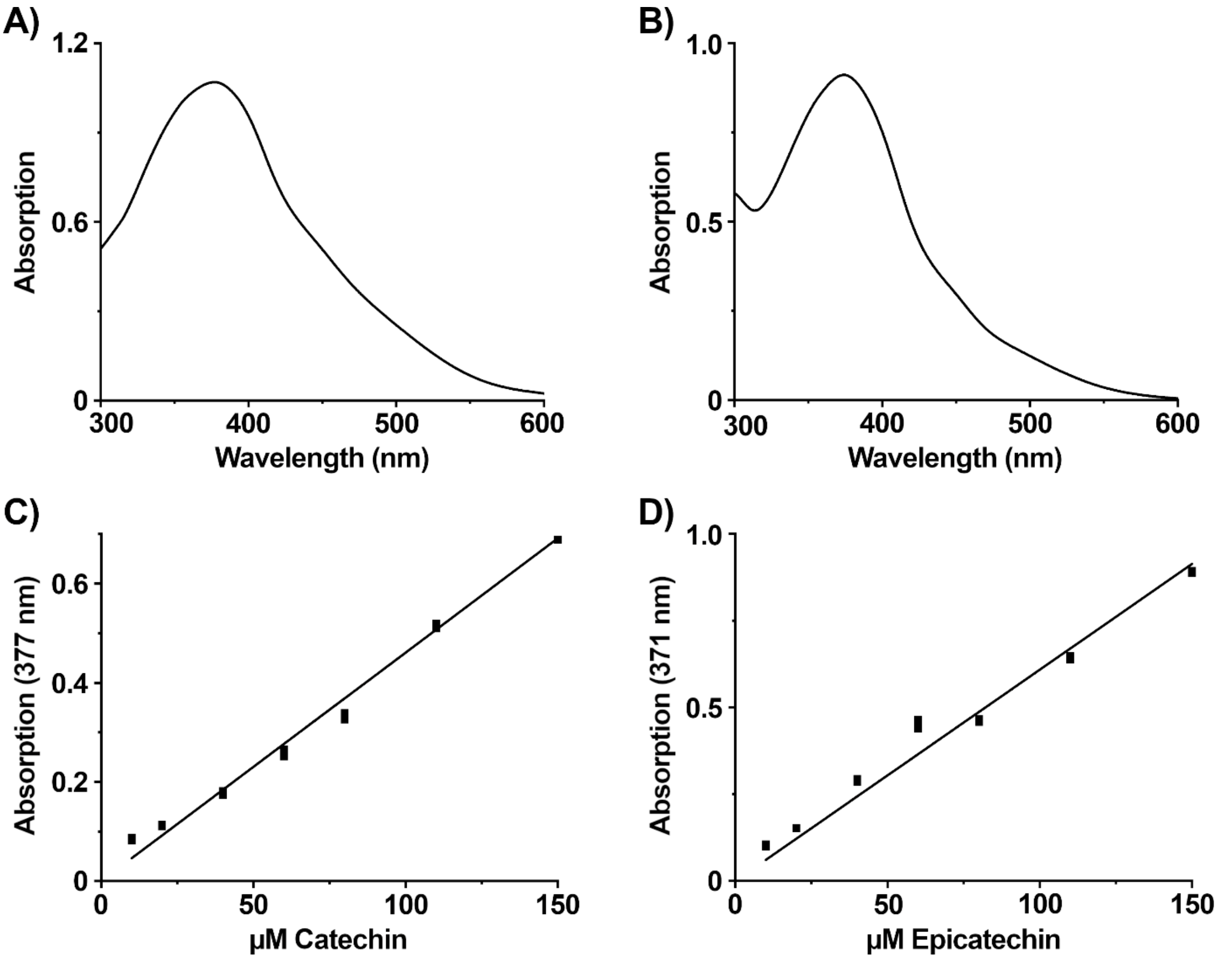

Figure S10. Determination of molar extinction coefficients. A & **B**: 265 µM (catechin, A) and 165 µM (epicatechin, B) substrate have respectively been oxidized in 50 mM TRIS – HCl pH 7.0 using 100 µg *Can*STYR. The UV/Vis spectra have been recorded on a Shimadzu 1800 photometer at 22 °C after a steady-state spectrum has been reached. **C** & **D**: Different molarities of catechin (A) and epicatechin (B) have been oxidized using 100 µg *Can*STYR. The absorption values have been recorded after a steady-state spectrum has been reached. The Figure has been created using OriginPro 8 and GIMP 2.10.18 (https://www.gimp.org).

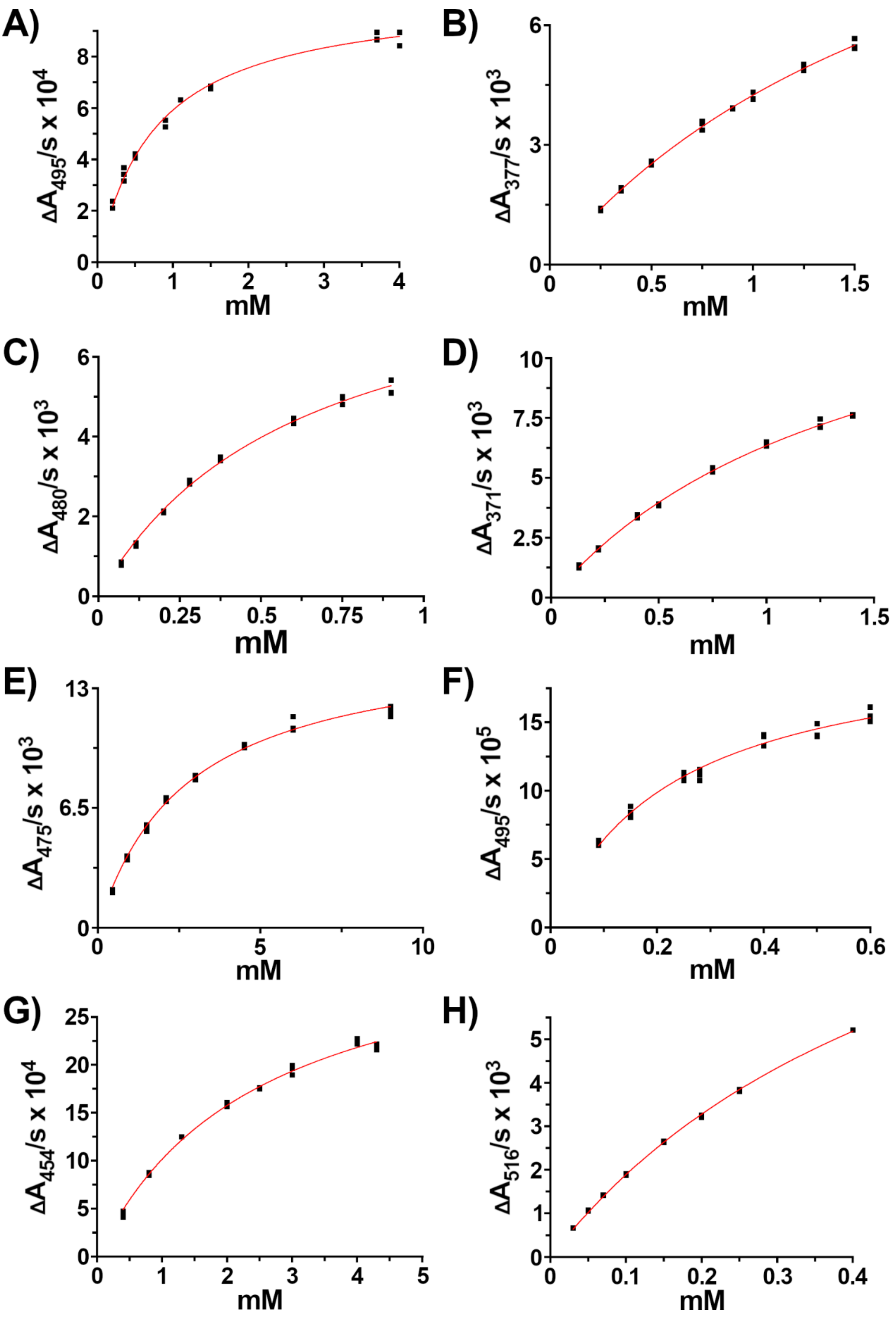

A)
ΔA495/s x 10^4
mM
B)
ΔA377/s x 10^3
mM
C)
ΔA480/s x 10^3
mM
D)
ΔA371/s x 10^3
mM
E)
ΔA475/s x 10^3
mM
F)
ΔA495/s x 10^5
mM
G)
ΔA454/s x 10^4
mM
H)
ΔA516/s x 10^3
mM

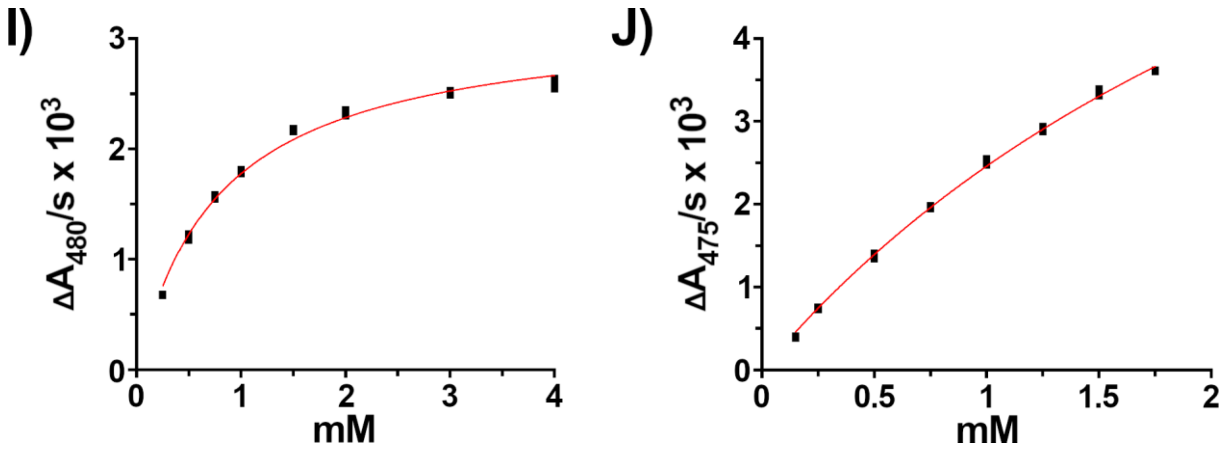

Figure S11. Non-linear curve fitting for active substrates with *Can*STYR. A = caffeic acid, B = catechin, C = dopamine, D = epicatechin, E = *L*-DOPA, F = *p*-coumaric acid, G = protocatechuic acid, H = taxifolin, I = tyramine, J = *L*-tyrosine. A detailed description of the experimental setup is provided in the Materials and Methods section. The amounts of *Can*STYR used for each respective measurement are listed in Table S4. Activities plotted represent the maximum reaction rates reached after overcoming the lag period (1 – 5 minutes) for monophenols. For diphenols, maximum reaction rates are reached immediately. The Figure has been created using OriginPro 8 and GIMP 2.10.18 (https://www.gimp.org).

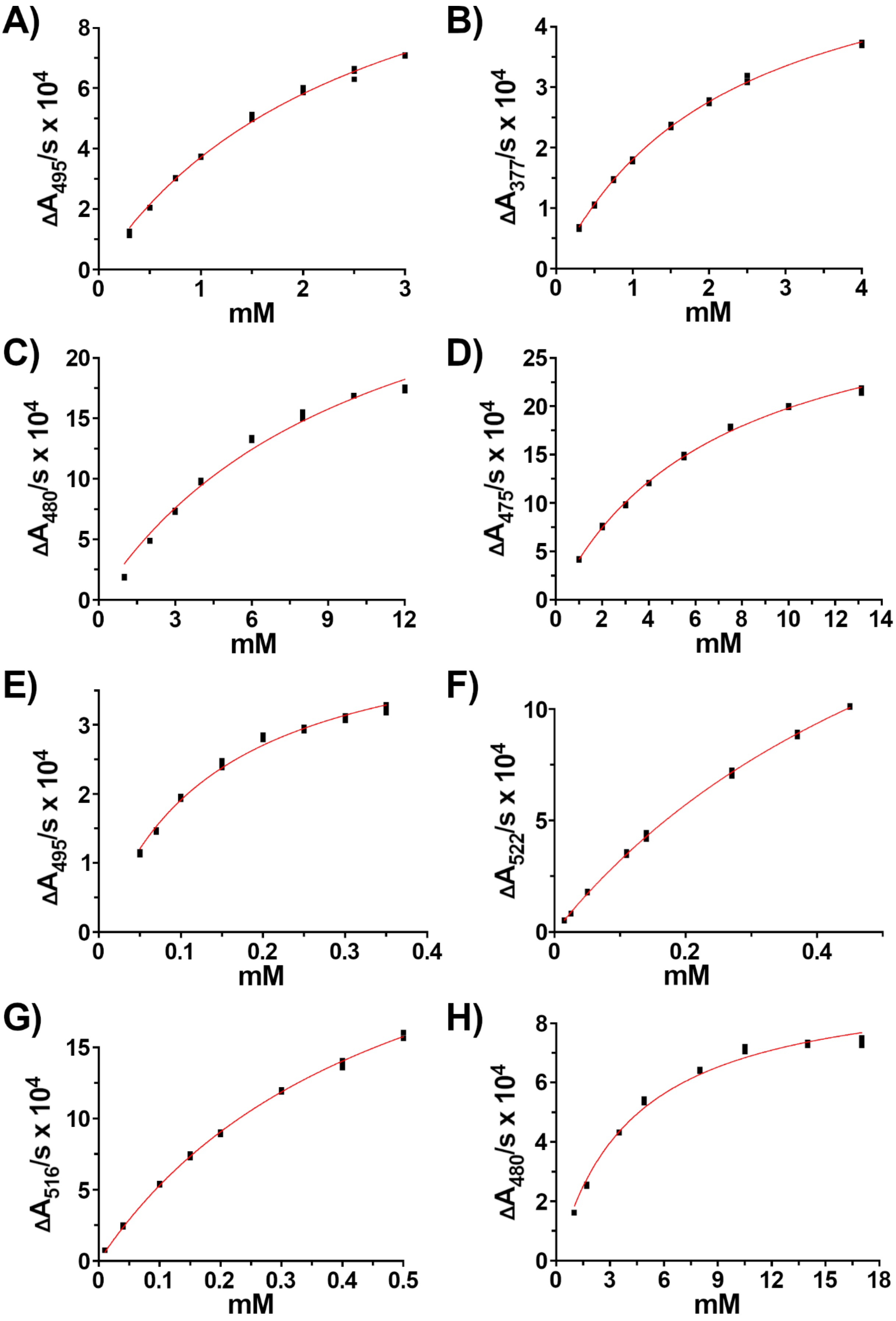
A)
ΔA_{495}/s x 10^4
mM
B)
ΔA_{377}/s x 10^4
mM
C)
ΔA_{480}/s x 10^4
mM
D)
ΔA_{475}/s x 10^4
mM
E)
ΔA_{495}/s x 10^4
mM
F)
ΔA_{522}/s x 10^4
mM
G)
ΔA_{516}/s x 10^4
mM
H)
ΔA_{480}/s x 10^4
mM

I)

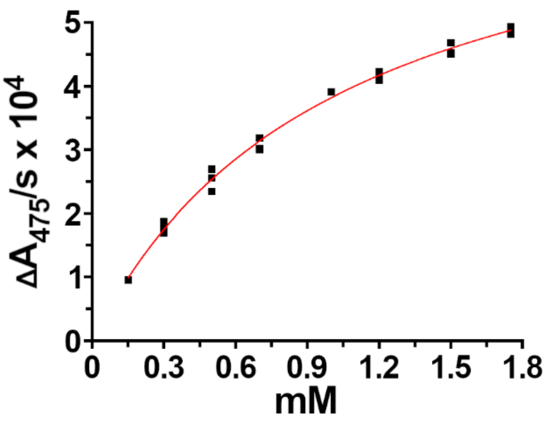

Figure S12. Non-linear curve fitting for active substrates with *CabS*TYR. A = caffeic acid, B = catechin, C = dopamine, D = *L*-DOPA, E = *p*-coumaric acid, F = quercetin, G = taxifolin, H = tyramine, I = *L*-tyrosine. A detailed description of the experimental setup is given in the Materials and Methods section. The amounts of *CabS*TYR used for each respective measurement are listed in Table S4. Activities plotted represent the maximum reaction rates reached after overcoming the lag period (1 – 5 minutes) for monophenols. For diphenols, maximum reaction rates are reached immediately. The Figure has been created using OriginPro 8 and GIMP 2.10.18 (https://www.gimp.org).

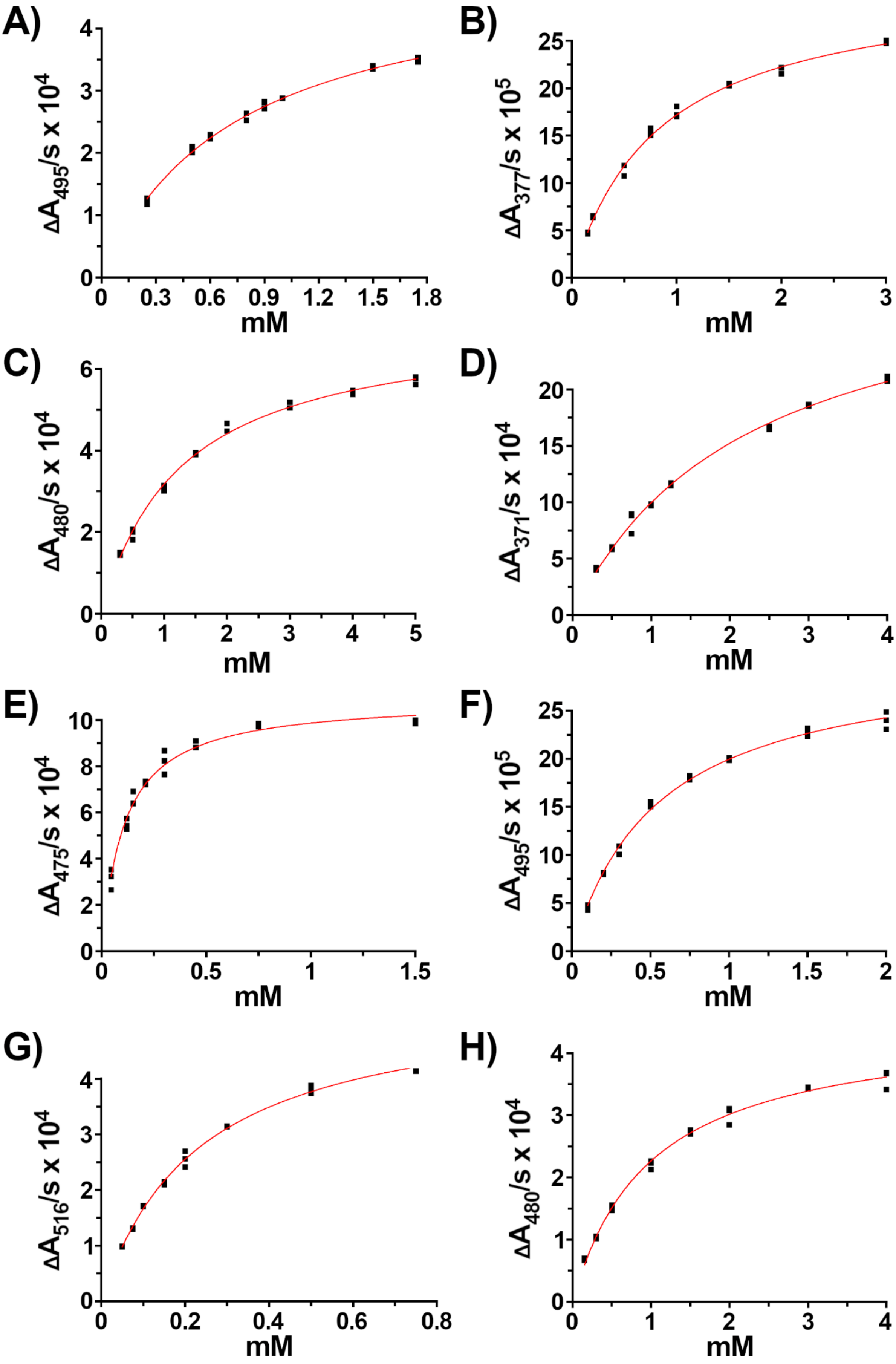
A)
ΔA495/s x 10^4
mM
B)
ΔA377/s x 10^5
mM
C)
ΔA480/s x 10^4
mM
D)
ΔA371/s x 10^4
mM
E)
ΔA475/s x 10^4
mM
F)
ΔA495/s x 10^5
mM
G)
ΔA516/s x 10^4
mM
H)
ΔA480/s x 10^4
mM

I)

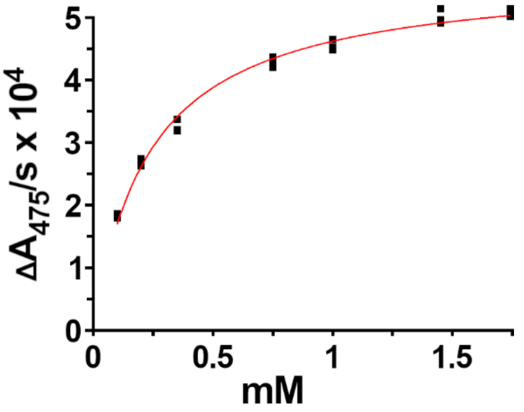

Figure S13. Non-linear curve fitting for active substrates with *Sin*ATYR. A = caffeic acid, B = catechin, C = dopamine, D = epicatechin, E = *L*-DOPA, F = *p*-coumaric acid, G = taxifolin, H = tyramine, I = *L*-tyrosine. A detailed description of the experimental setup is given in the Materials and Methods section. The amounts of *Sin*ATYR used for each respective measurement are listed in Table S4. Activities plotted represent the maximum reaction rates reached after overcoming the lag period (1 – 5 minutes) for monophenols. For diphenols, maximum reaction rates are reached immediately. The Figure has been created using OriginPro 8 and GIMP 2.10.18 (https://www.gimp.org).

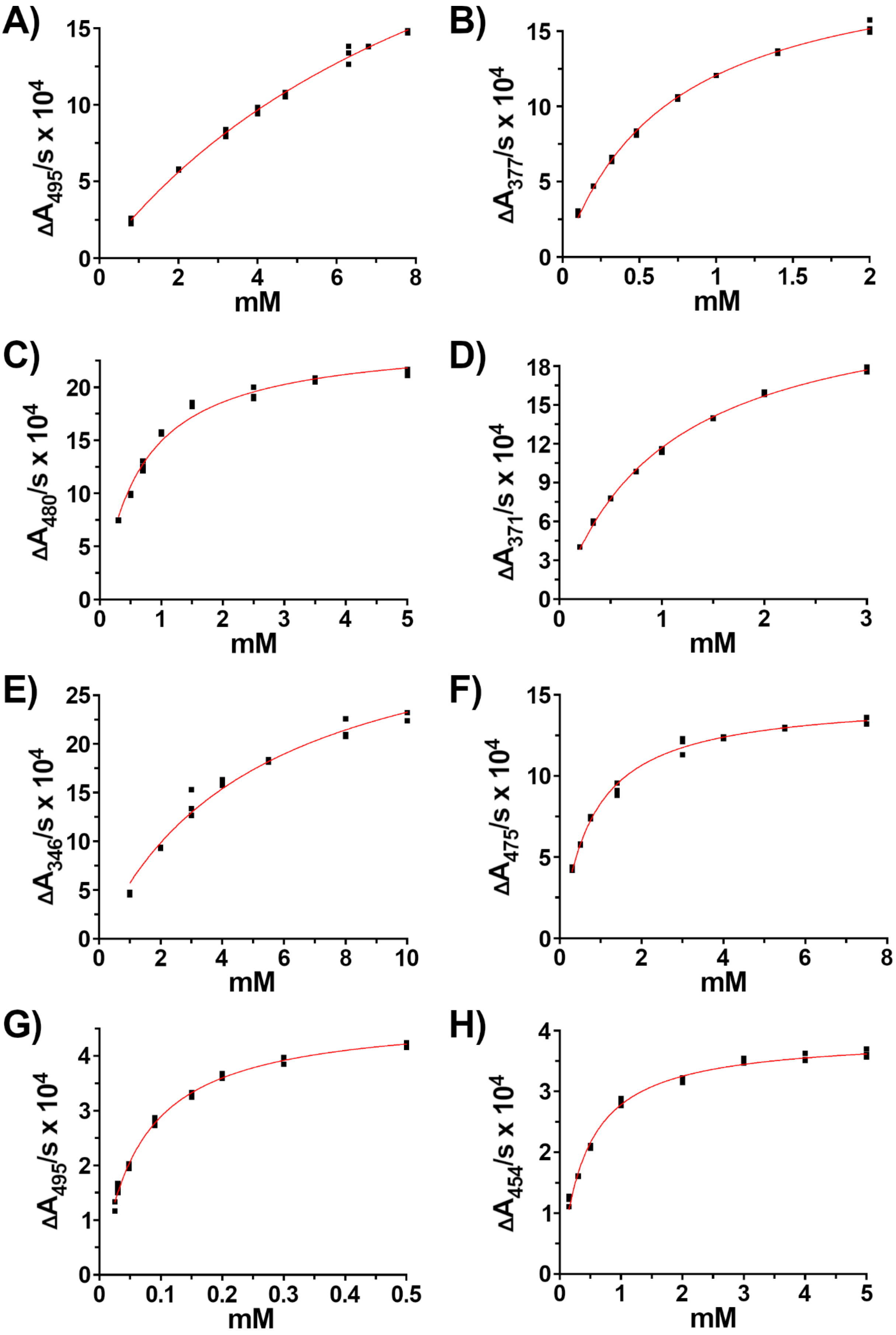

A)
15
10
5
0
0
2
4
6
8
ΔA495/s x 10^4
mM
B)
15
10
5
0
0
0.5
1
1.5
2
ΔA377/s x 10^4
mM
C)
20
15
10
5
0
0
1
2
3
4
5
ΔA480/s x 10^4
mM
D)
18
15
12
9
6
3
0
0
1
2
3
ΔA371/s x 10^4
mM
E)
25
20
15
10
5
0
0
2
4
6
8
10
ΔA346/s x 10^4
mM
F)
15
10
5
0
0
2
4
6
8
ΔA475/s x 10^4
mM
G)
4
3
2
1
0
0
0.1
0.2
0.3
0.4
0.5
ΔA495/s x 10^4
mM
H)
4
3
2
1
0
0
1
2
3
4
5
ΔA454/s x 10^4
mM

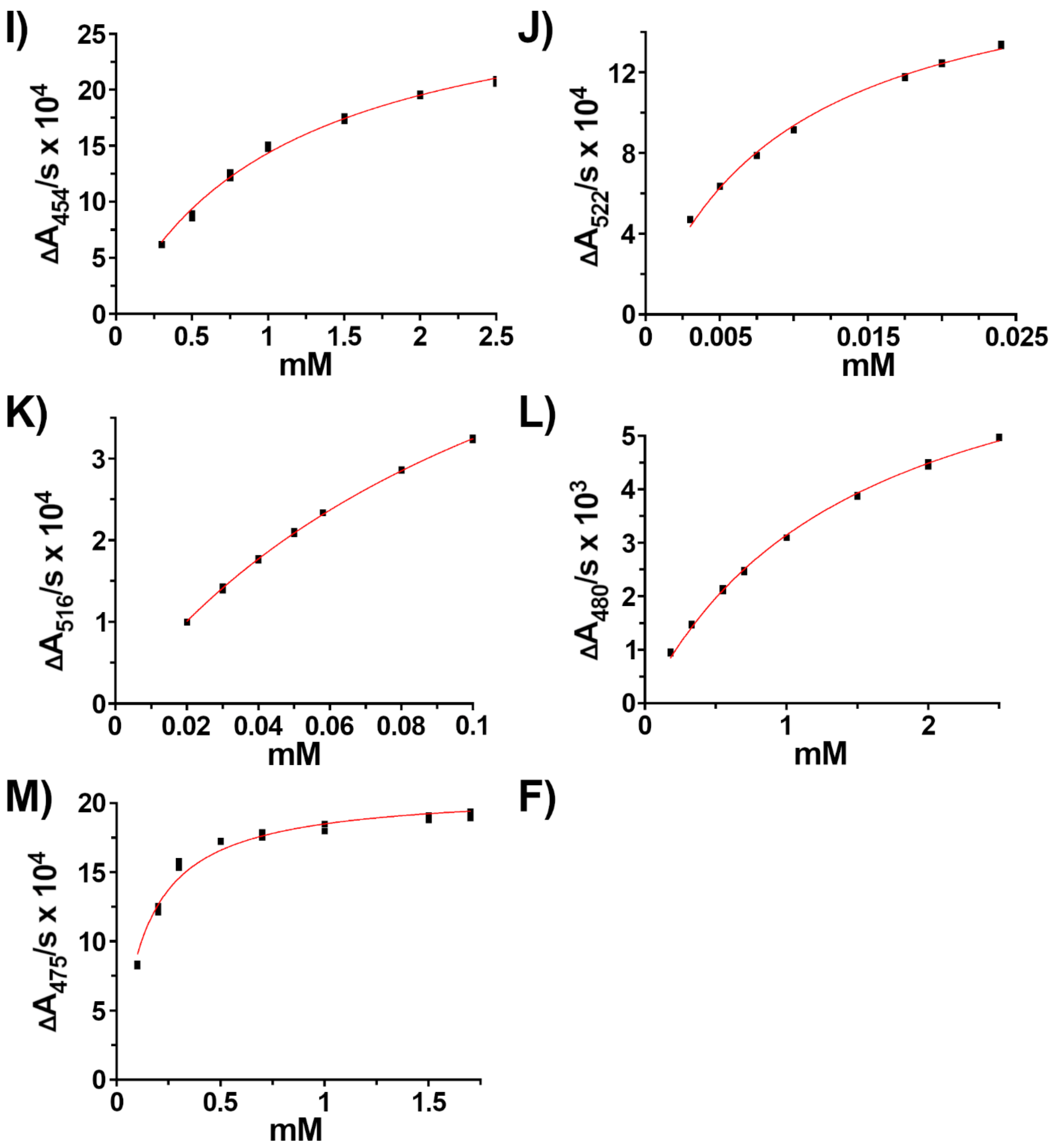

Figure S14. Non-linear curve fitting for active substrates with *Pse*STYR. A = caffeic acid, B = catechin, C = dopamine, D = epicatechin, E = gallic acid, F = *L*-DOPA, G = *p*-coumaric acid, H = *p*-hydroxybenzoic acid, I = protocatechuic acid, J = quercetin, K = taxifolin, L = tyramine, M = *L*-tyrosine. A detailed description of the experimental setup is given in the Materials and Methods section. The amounts of *Pse*STYR used for each respective measurement are listed in Table S4. Activities plotted represent the maximum reaction rates reached after overcoming the lag period (1 – 5 minutes) for monophenols. For diphenols, maximum reaction rates are reached immediately. The Figure has been created using OriginPro 8 and GIMP 2.10.18 (https://www.gimp.org).

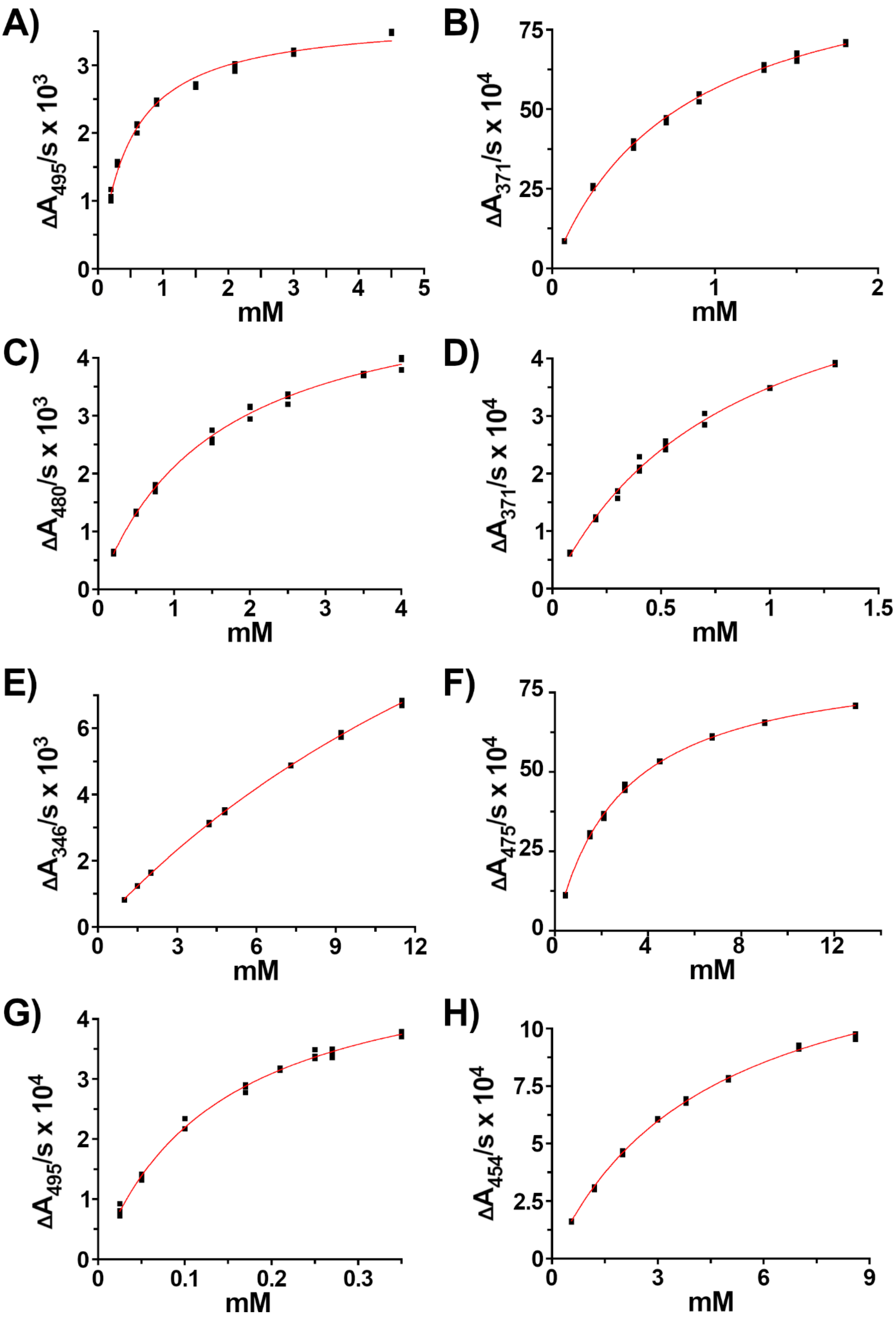
A)
ΔA495/s x 10^3
mM
B)
ΔA371/s x 10^4
mM
C)
ΔA480/s x 10^3
mM
D)
ΔA371/s x 10^4
mM
E)
ΔA346/s x 10^3
mM
F)
ΔA475/s x 10^4
mM
G)
ΔA495/s x 10^4
mM
H)
ΔA454/s x 10^4
mM

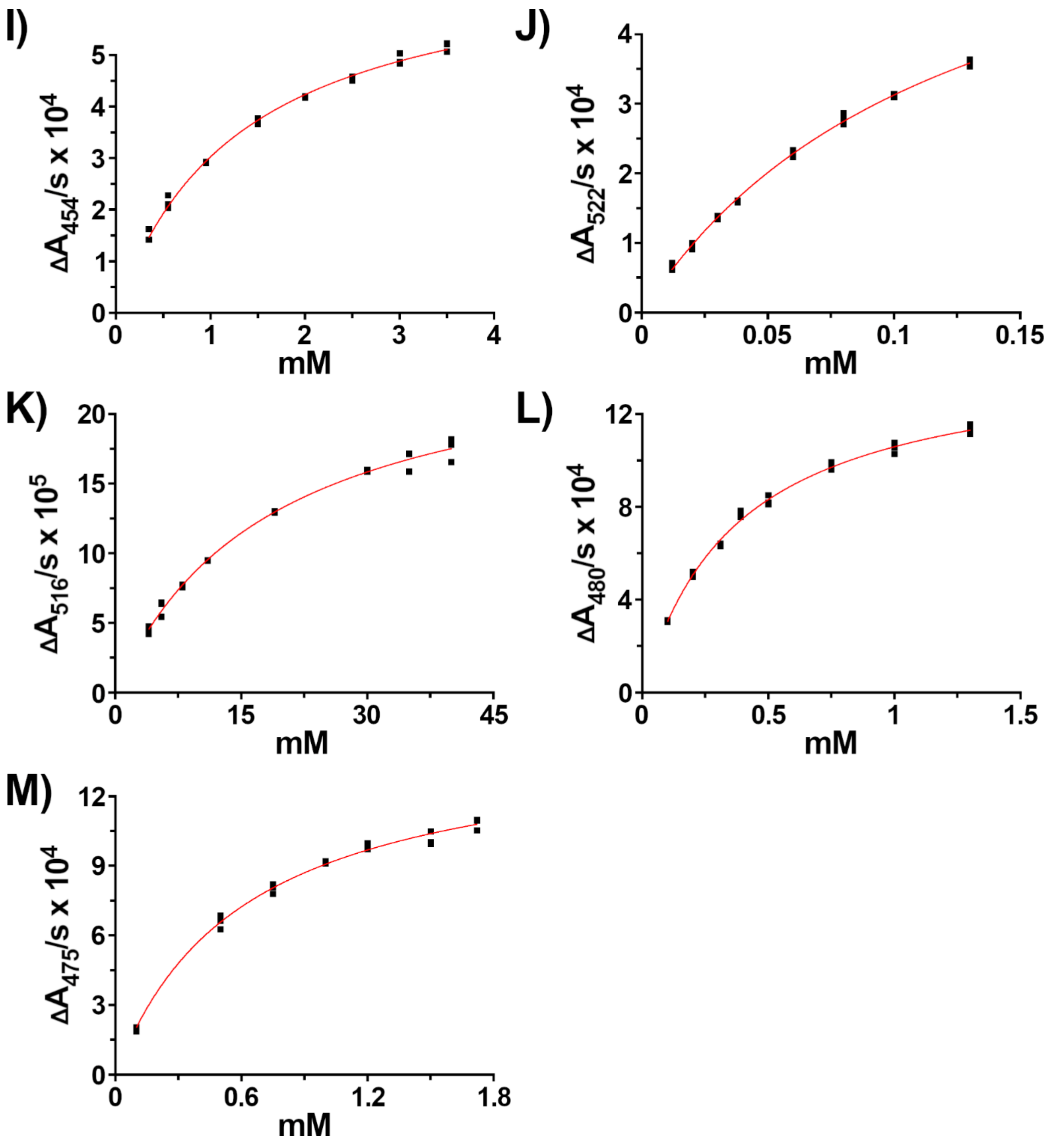

Figure S15. Non-linear curve fitting for active substrates with *Chr*STYR. A = caffeic acid, B = catechin, C = dopamine, D = epicatechin, E = gallic acid, F = *L*-DOPA, G = *p*-coumaric acid, H = *p*-hydroxybenzoic acid, I = protocatechuic acid, J = quercetin, K = taxifolin, L = tyramine, M = *L*-tyrosine. A detailed description of the experimental setup is given in the Materials and Methods section. The amounts of *Chr*STYR used for each respective measurement are listed in Table S4. Activities plotted represent the maximum reaction rates reached after overcoming the lag period (1 – 5 minutes) for monophenols. For diphenols, maximum reaction rates are reached immediately. The Figure has been created using OriginPro 8 and GIMP 2.10.18 (https://www.gimp.org).

References.

(1) Faccio, G.; Kruus, K.; Saloheimo, M.; Thöny-Meyer, L. Bacterial Tyrosinases and Their Applications. *Process Biochem.* **2012**, *47*, 1749–1760. https://doi.org/10.1016/j.procbio.2012.08.018.

(2) Claus, H.; Decker, H. Bacterial Tyrosinases. *Syst. Appl. Microbiol.* **2006**, *29*, 3–14. https://doi.org/10.1016/j.syapm.2005.07.012.

(3) Pretzler, M.; Bijelic, A.; Rompel, A. Heterologous Expression and Characterization of Functional Mushroom Tyrosinase (*Ab*PPO4). *Sci. Rep.* **2017**, *7*, 1810. https://doi.org/10.1038/s41598-017-01813-1.

(4) Gasteiger, E.; Hoogland, C.; Gattiker, A.; Duvaud, S.; Wilkins, M. R.; Appel, R. D.; Bairoch, A. The Proteomics Protocols Handbook. **2005**, 571–608. https://doi.org/10.1385/1592598900.

(5) Muñoz, J. L.; García-Molina, F.; Varón, R.; Rodriguez-Lopez, J. N.; García-Cánovas, F.; Tudela, J. Calculating Molar Absorptivities for Quinones: Application to the Measurement of Tyrosinase Activity. *Anal. Biochem.* **2006**, *351*, 128–138. https://doi.org/10.1016/j.ab.2006.01.011.

(6) Trott, O.; Olson, A. Autodock Vina: Improving the Speed and Accuracy of Docking. *J Comput Chem* **2010**, *31*, 455–461. https://doi.org/10.1002/jcc.21334.AutoDock.

(7) Jumper, J.; Evans, R.; Pritzel, A.; Green, T.; Figurnov, M.; Ronneberger, O.; Tunyasuvunakool, K.; Bates, R.; Žídek, A.; Potapenko, A.; Bridgland, A.; Meyer, C.; Kohl, S. A. A.; Ballard, A. J.; Cowie, A.; Romera-Paredes, B.; Nikolov, S.; Jain, R.; Adler, J.; Back, T.; Petersen, S.; Reiman, D.; Clancy, E.; Zielinski, M.; Steinegger, M.; Pacholska, M.; Berghammer, T.; Bodenstein, S.; Silver, D.; Vinyals, O.; Senior, A. W.; Kavukcuoglu, K.; Kohli, P.; Hassabis, D. Highly Accurate Protein Structure Prediction with AlphaFold. *Nature* **2021**, *596*, 583–589. https://doi.org/10.1038/s41586-021-03819-2.

(8) Varadi, M.; Anyango, S.; Deshpande, M.; Nair, S.; Natassia, C.; Yordanova, G.; Yuan, D.; Stroe, O.; Wood, G.; Laydon, A.; Zídek, A.; Green, T.; Tunyasuvunakool, K.; Petersen, S.; Jumper, J.; Clancy, E.; Green, R.; Vora, A.; Lutfi, M.; Figurnov, M.; Cowie, A.; Hobbs, N.; Kohli, P.; Kleywegt, G.; Birney, E.; Hassabis, D.; Velankar, S. AlphaFold Protein Structure Database: Massively Expanding the Structural Coverage of Protein-Sequence Space with High-Accuracy Models. *Nucleic Acids Res.* **2022**, *50*, D439–D444. https://doi.org/10.1093/nar/gkab1061.

(9) Stevenson, F. J. Humus Chemistry: Genesis, Composition, Reactions, 2nd Edition. *Wiley, New York.*; **1994**. https://doi.org/10.1021/ed072pA93.6.

(10) Panis, F.; Rompel, A. Identification of the Amino Acid Position Controlling the Different Enzymatic Activities in Walnut Tyrosinase Isoenzymes (*Jr*PPO1 and *Jr*PPO2). *Sci. Rep.* **2020**, *10*, 10813. https://doi.org/10.1038/s41598-020-67415-6.

(11) Goldfeder, M.; Kanteev, M.; Isaschar-Ovdat, S.; Adir, N.; Fishman, A. Determination of Tyrosinase Substrate-Binding Modes Reveals Mechanistic Differences between Type-3 Copper Proteins. *Nat. Commun.* **2014**, *5*, 4505. https://doi.org/10.1038/ncomms5505.

(12) López-Serrano, D.; Sanchez-Amat, A.; Solano, F. Cloning and Molecular Characterization of a SDS-Activated Tyrosinase from *Marinomonas mediterranea*. *Pigment Cell Res.* **2002**, *15*, 104–111. https://doi.org/10.1034/j.1600-0749.2002.1o068.x.

(13) Sellés-Marchart, S.; Casado-Vela, J.; Bru-Martínez, R. Effect of Detergents, Trypsin and Unsaturated Fatty Acids on Latent Loquat Fruit Polyphenol Oxidase: Basis for the Enzyme's Activity Regulation. *Arch. Biochem. Biophys.* **2007**, *464*, 295–305. https://doi.org/10.1016/j.abb.2007.04.023.

(14) Gandía-Herrero, F.; Jiménez-Atiénzar, M.; Cabanes, J.; García-Carmona, F.; Escribano, J. Differential Activation of a Latent Polyphenol Oxidase Mediated by Sodium Dodecyl Sulfate. *J. Agric. Food Chem.* **2005**, *53*, 6825–6830. https://doi.org/10.1021/jf050505e.

(15) Kampatsikas, I.; Bijelic, A.; Pretzler, M.; Rompel, A. A Peptide Inducing Self-Cleavage Reaction Initiates the Activation of Tyrosinase. *Angew. Chem. Int. Ed.* **2019**, *58*, 7475–7479. https://doi.org/10.1002/anie.201901332. and Eine peptidvermittelte Selbstspaltungsreaktion initiiert die Tyrosinaseaktivierung. *Angew. Chem. Int. Ed.* **2019**, *58*, 7475–7479. https://doi.org/10.1002/ange.201901332.

(16) Ito, S.; Sugumaran, M.; Wakamatsu, K. Chemical Reactivities of Ortho-Quinones Produced in Living Organisms: Fate of Quinonoid Products Formed by Tyrosinase and Phenoloxidase Action on Phenols and Catechols. *Int. J. Mol. Sci.* **2020**, *21*, 6080. https://doi.org/10.3390/ijms21176080.

(17) Klavins, M.; Purmalis, O. Properties and Structure of Raised Bog Peat Humic Acids. *J. Mol. Struct.* **2013**, *1050*, 103–113. https://doi.org/10.1016/j.molstruc.2013.07.021.

(18) Klavins, M.; Purmalis, O. Characterization of Humic Acids from Raised Bog Peat. *Latv. J. Chem.* **2014**, *52*, 83–97. https://doi.org/10.2478/ljc-2013-0010.

(19) Vasilevich, R.; Lodygin, E.; Abakumov, E. The Molecular Composition of Humic Acids in Permafrost Peats in the European Arctic as Paleorecord of the Environmental Conditions of the Holocene. *Agronomy* **2022**, *12*, 2053. https://doi.org/10.3390/agronomy12092053.
